# Identification and analysis of miRNAs differentially expressed in male and female *Trichosanthes kirilowii* maxim

**DOI:** 10.1186/s12864-023-09178-8

**Published:** 2023-02-21

**Authors:** Xiu-qin Hu, Han Song, Na Li, Chun-xiang Hao, Bo Zhang, Xin-peng Li, Jie Xin, Yong-qing Zhang

**Affiliations:** 1 Medical College, Linyi University, Lin’yi, 276000 China; 2 School of Pharmacy, Shandong University of Traditional Chinese Medicine, Ji’nan, 250355 China

**Keywords:** *TK*, Sex differentiation, Transcriptome and sRNA sequencing, miRNA

## Abstract

*Trichosanthes kirilowii* Maxim. (*TK*) is a dioecious plant in the Cucurbitaceae family of which different sexes have separate medicinal uses. We used Illumina high-throughput sequencing technology to sequence miRNAs from male and female flower buds of *TK*. We performed bioinformatics analysis, miRNA identification, and target gene prediction on the data obtained from sequencing, and association analysis was performed in combination with the results of a previous transcriptome sequencing study. As a result, there were 80 differentially expressed miRNAs (DESs) between the female and male plants (48 upregulated and 32 downregulated in female plants). Moreover, 27 novel miRNAs in DESs were predicted to have 282 target genes, and 51 known miRNAs were predicted to have 3418 target genes. By establishing a regulatory network between miRNAs and target genes, 12 core genes were screened, including 7 miRNAs and 5 target genes. Among them, tkmiR157a-5p, tkmiR156c, tkmiR156_2, and tkmiR156k_2 jointly target the regulation of *tkSPL18* and *tkSPL13B.* These two target genes are specifically expressed in male and female plants, respectively, and are involved in the biosynthesis process of BR, which is closely related to the sex differentiation process of *TK.* The identification of these miRNAs will provide a reference for the analysis of the sex differentiation mechanism of *TK*.

**Supplementary Information:**

The online version contains supplementary material available at 10.1186/s12864-023-09178-8.

## Introduction

*Trichosanthes kirilowii* Maxim. (*TK*) is a member of the Cucurbitaceae family, and is used to treat cough, phlegm, constipation, and various carbuncles by clearing heat and removing phlegm, moistening the lung and relieving cough, broadening the chest and dispersing knots, and moistening the intestines [1]. *TK* is a typical dioecious plant, which plays an important role in elucidating the mechanism of plant sex determination and evolution. The male and female plants of *TK* have different medicinal values. A large number of female plants (and a small number of male plants) are required for harvesting seeds and fruits; male plants are required for harvesting roots. At present, there are two propagation methods: vegetative propagation using rhizomes and sexual propagation using seeds. Rhizome propagation can control the sex of the plant but the reproduction coefficient is low and a large amount of root is consumed; seed propagation can greatly improve the planting efficiency but the sex ratio of the plant is out of balance and cannot be controlled (male to female ratio 3:7) [2]. Therefore, it is important to explore the mechanism of sex determination in *TK*.

Sex differentiation is manifested in the floral organs, and the development rate of *TK* male flowers is faster than that of female flowers. The differentiation process of the male flowers of unisexual *TK* can be divided into six periods, and the whole development process only sees the differentiation and growth of the stamen primordia. The differentiation process of the female flowers of *TK* as “hermaphroditic flowers” can be divided into seven periods, and there is a stage of common development of pistil and stamen, and stamen development is halted at the later stage [3]. Therefore, the study of the formation of floral organs in *TK* is very important for understanding the sex differentiation mechanism.

MicroRNAs (miRNAs) are biologically endogenous non-coding RNAs of 18–24 nt in length and are an important class of gene expression regulators [4]. The main regulatory mechanism of miRNAs is to act by regulating downstream target genes [5]. By complementary pairing with mRNAs, they target mRNAs for degradation or inhibit their translation at the post-transcriptional level [6]. They affect protein expression and thus regulate the physiological functions of the body [7]. miRNAs are widely involved in the regulation of various biological processes in plant growth and development, including cell development and differentiation, biotic and abiotic stress responses, maturation, and senescence [8]. Therefore, miRNAs have received much attention in plant research. Existing studies have confirmed that miRNAs play an important role in flower development [9]. In addition, some highly conserved miRNAs can regulate the development of floral organs [10]. miRNAs affecting flower development have been isolated and identified from *Arabidopsis thaliana*, *Zea mays*, *Oryza sativa*, *Petunia*, and other model plants [11–14]. For example, miR159, which targets *MYB*, can affect anther development in *Arabidopsis* [15]. miR156 targets *SPL* and controls flowering transition in *Arabidopsis* [16]. Some miRNAs have been shown to be involved in the process of sex differentiation in plants. For example, miRl72e, encoded by the *ts4* gene in *Z. mays*, acts on an *APETALA2* homologous transcription factor (TF) gene, *idsl*, to suppress pistil development in male flowers and promote male flower morphogenesis [17]. The miRNA encoded by the Y chromosome-specific gene *OGI* in persimmon acts on the autosomal *MeGI* gene to prevent pollen sterility, which can be produced by male plants to suppress *MeGI* gene expression and normal pollen development in male flowers, but not in female plants, where pollen abortion occurs [18]. They share a similar sex determination pattern; one sex-determination gene encodes a miRNA to repress the expression of another sex-determination gene, indicating that miRNAs play a crucial role in the gene regulatory network of plant sex differentiation [19]. In this study, high-throughput small RNA (sRNA) sequencing was performed on *TK* male and female flower bud samples and combined with transcriptome information analysis to screen *TK* flower organ differentiation-related miRNAs, laying the foundation for further in-depth study of the sex differentiation mechanism in *TK*.

## Materials and methods

### Sample collection

Previous studies have shown that 2 mm is the critical period for sexual differentiation of *TK*, so the material of this study selected 2 mm male and female *TK* buds [3]. Three biological replicates were established with a total of six samples. Female flower buds were named F1, F2, and F3, while male flower buds were named M1, M2, and M3. The samples were wrapped in tin foil, snap-frozen in liquid nitrogen, and stored at − 80 °C until analysis.

### mRNA and sRNA library construction and sequencing

The flower bud samples were sent to The Beijing Genomics Institute (BGI, Shenzhen, China) for sRNA sequencing and transcriptome sequencing. For details on mRNA library construction, sequencing, and transcriptome data used in this article, please refer to the published articles of our group [20]. Total RNA was extracted from each sample using TRIzol (TAKARA, 9109). The concentration, quality, and integrity of the extracted total RNA were checked using an Agilent 2100 Bioanalyzer. The sRNA libraries were constructed using BGISEQ-500 sequencing technology [21].

### Basic analysis of sRNA sequencing tags

The raw tags obtained by sequencing were analyzed as follows. To obtain high-quality tags, the low-quality tags with base mass value < 10 and base number ≤ 4 or base mass value < 13 and base number ≤ 6 were filtered out with SOAPnuke software (v1.4.0, −l 15 -q 0.2 -n 0.1). The tags with 5′ junction contamination and no 3′ junction, the tags without insert fragment, the tags containing polyA, and the tags with < 18 nt or > 30 nt were removed to obtain the clean tags. Based on the obtained clean tags, the length distribution of tags was determined.

### Identification of known and novel miRNAs

Using AASRA [22] and cmsearch [23] software, the tags were analyzed with the miRBase [24], Rfam [25], siRNA, piRNABank, and other databases for comparison and annotation. By comparing the *TK* transcriptome with the software AASRA, duplicate tags and reads from mRNA degradation fragments were removed to obtain unique reads. In each of the above annotations, there may be different annotation information at the same time. To obtain a unique annotation for each unique sRNA, the sRNAs were annotated in the following order of priority: MiRbase > piRNAbank > snoRNA (human/plant) > Rfam > other sRNAs. The rRNAs, scRNAs, snoRNAs, snRNAs, and tRNAs in the samples were removed. Unique reads were identified as known miRNAs by comparing them with the mature miRNAs already included in the miRBase database and selecting the mature bodies and precursor information of the miRNAs. The remaining unknown part was annotated based on the precursors of miRNAs capable of forming secondary hairpin structures, using the miRNA prediction software miRDeep2 [26] (for animals) and miRA [27] (for plants) to predict novel miRNAs. The screening criteria were to be able to form a stem-loop secondary structure and have the minimum free energy (< 0.2 kcal/mol). Precursor secondary structures and MEF value were produced using the RNAFOLD (Default parameters) software. The known miRNAs were combined with the novel miRNAs to obtain the full miRNAs.

### miRNA expression analysis and target gene prediction

The expression profiles of all miRNAs were obtained by combining the expression of miRNAs in each sample. Differentially expressed miRNAs (DESs; fold change > 2 and Q ≤ 0.001) were screened (DEGseq) [28] based on MA-plot [29]. The gene clustering heatmap was plotted by TBtools. Target genes were predicted using psRobot (−gl 17 -p 8 -gn 1) [30], TAPIR (score 5 mfe_ratio 0.6) [31], and TargetFinder (c 4) [32]. Filtering was performed with appropriate filtering conditions such as free energy and score value, and results supported by at least two target gene prediction software programs were selected.

### Validation of miRNA and target gene

We used the modified 5′ RACE method to validate miRNA and target gene pairs. Briefly, the total RNA of male and female flower buds was reverse-transcribed into first-strand cDNA using the gene-specific primer SP1, and then add homopolymeric A-tail to 3’end of first-strand cDNA using terminal transferase and dATP (Roche, 03353621001). The first round of PCR amplification was performed using Oligo dT-Anchor Primer and specific primer SP2. Amplify 1 uL of the first PCR product using the PCR Anchor Primer and a nested gene-specific primer 3 (SP3) in a second PCR. The above primer sequences were shown in Additional file 1. The first and second rounds of PCR amplification conditions were as follows: 94 °C for 2 min, followed by 35 cycles at 94 °C for 10 s, 55-60 °C for 30 s, and 72 °C for 40 s with a final extension of 10 min at 72 °C (From the 11th cycle, the extension time was added by an additional 20 s). PCR products were separated on 1% agarose gel, distinct bands were gel purified, ligated into the pEASY-blunt Zero cloning vector (TransGen, CB501), and sequenced using M13 primer.

### Functional enrichment analysis of target genes for DESs

The GO and KEGG analyses were performed on the target genes of differentially expressed miRNAs (DESs). GO terms were subjected to functional enrichment analysis (*P* ≤ 0.05) to determine the major biological functions exercised by the target genes corresponding to DESs. The most significant biochemical metabolic pathways and signal transduction pathways in which the target genes of DESs were involved were then determined (Q ≤ 0.05).

### RT-qPCR verifies sRNA sequencing results

RNAiso (TAKARA, 9753A) was used to extract small RNA, and cDNA was synthesized according to the Mir-X™ miRNA FirstStrand Synthesis instructions (TAKARA, 638313). Forward primers were designed in Primer Premier5 software (Additional file 1), and reverse primers are generic primers for TB Green® Premix Ex Taq™ II (TAKARA, RR820A). The reaction program: 95 °C for 30 s, 40 cycles at 95°Cfor 5 s, and 60°Cfor 30 s. Using *U6* as an internal control, the 2^-ΔΔCT^ method calculated the relative quantification of gene expression.

### Combined transcriptome and sRNA sequencing analysis

Based on the expression levels of the two omics, further analyze the relationship between miRNA and its target genes. According to the expression levels and miRNA–target relationships in different samples, we calculated their Pearson correlation coefficients using the R package, where correlation coefficients with absolute values of > 0.6 were considered to be correlated. Based on the correlation coefficients and target relationships, we classified the results for each group of differences (positive and negative correlations): (1) miRNAs were negatively correlated with target genes if the correlation coefficients of miRNAs and target genes were negative and the fold change of miRNAs and target genes was one positive and one negative inside the same group; and (2) miRNAs were positively correlated with target genes if the correlation coefficients between miRNAs and target genes were positive and the fold change of miRNAs and target genes in the same group were both positive or both negative. Enrichment analysis of differentially co-expressed genes was performed, and the results of each differential group were classified (positive and negative correlation) based on correlation coefficients and target relationships and then subjected to GO and KEGG analysis.

## Results

### Sequence analysis of sRNA sequencing

**Fig. 1:**
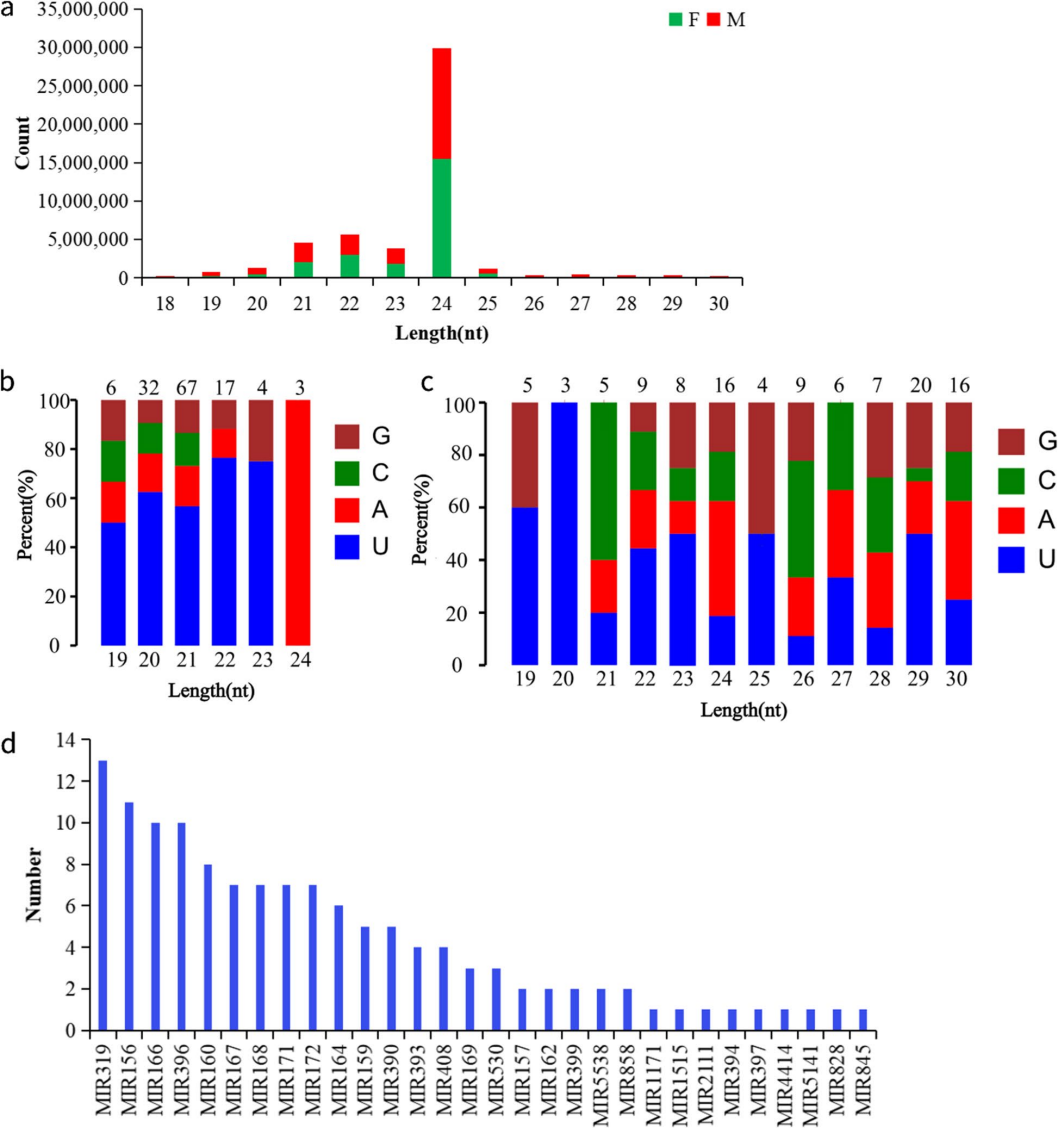
Characterization of sRNA-Seq data. **a** Length distribution of sRNAs from female and male flowers. **b** A Nucleotide bias analysis of known miRNAs. **c** Nucleotide bias analysis of novel miRNAs. **d** Number of members identified in the 30 conserved miRNA families

The raw tags were obtained in F1, F2, F3, M1, M2, and M3 by BGISEQ-500 sequencing. After data filtering, the 5′ end and 3′ end junctions, contaminating tags, and low-quality tags were removed, and finally, the clean tags were retained (Table 1). The statistics of the distribution of tags of 18-30 bp in length showed that the highest proportions of 24 nt tags were found in male and female flower buds: 15,467,999 (62.22%) and 14,459,567 (58.92%), respectively. The next higher proportions were tags of 21, 22, and 23 nt in length (Fig. 1a).

**Table 1:** Statistical basic analysis of sRNA sequencing data

Sample name	Sequence type	Raw tag count	Low-quality tag count	Invalid adapter tag count	PolyA tag count	Short valid length tag	Clean tag count	Q20 of clean tag(%)	Percentage of clean tag(%)
F1	SE50	26,702,870	1,121,309	491,755.00	7090	222,332	24,860,384	99.30	90.10
F2	SE50	26,343,038	976,624	404,722.00	8713	198,084	24,754,895	99.30	93.97
F3	SE50	26,329,921	1,227,194	442,557.00	6452	221,466	24,432,252	99.20	92.79
M1	SE50	25,640,412	1,090,458	529,806.00	11,929	352,910	23,655,309	99.40	92.26
M2	SE50	25,090,813	649,863	648,044.00	14,661	513,151	24,265,094	99.30	93.00
M3	SE50	25,873,915	398,264	445,281.00	2726	190,373	24,837,271	99.20	95.99

F represents female samples, M represents male samples; 1, 2, and 3 represent different replicates, respectively

### Identification of known and novel miRNAs

By comparing with the known sRNA database, a total of 129 known miRNAs were identified in male and female flower buds after traversal annotation, and the remaining unknown part was predicted for novel sRNAs. In addition, by comparing with the mature body of known miRNAs in the miRBase database, the mature body and precursor information of miRNAs (known miRNAs) were obtained, and by hairpin structure prediction, a final total of 108 novel miRNAs were identified in both male and female flower buds. The length of novel miRNA precursors ranged from 60 to 1339 nt, and the MFE values obtained for these precursors ranged from − 21.20 kcal/mol to − 588.80 kcal/mol (Additional file 2). Mature and precursor sequences for all miRNAs were listed in Additional file 3.

Nucleotide bias analysis was performed for known and novel miRNAs. The results showed that among the known miRNAs, the frequency of U appearing at the beginning was higher for miRNAs between 19 and 23 nt in length, with an average proportion of more than 60%; meanwhile, the proportion of A for miRNAs of 24 nt in length was 100% (Fig. 1b). Among the novel miRNAs, the average proportion of U for 19 nt miRNAs was about 60.0%; the average proportion of U in 20 nt miRNAs was 100.0%; and the average percentage of C in 21 nt miRNAs was about 60.0% (Fig. 1c).

A total of 30 miRNA gene families were identified among the known miRNAs (Fig. 1d). The largest gene family was *MIR319*, containing 13 miRNA members, followed by *MIR156*, *MIR396*, and *MIR166*, containing 13, 11, and 11 family members, respectively. Most of the remaining gene families contained only one family member, such as *MIR1171*, *MIR1515*, *MIR2111*, and *MIR394*.

### Identification and target gene prediction of DESs

**Fig. 2:**
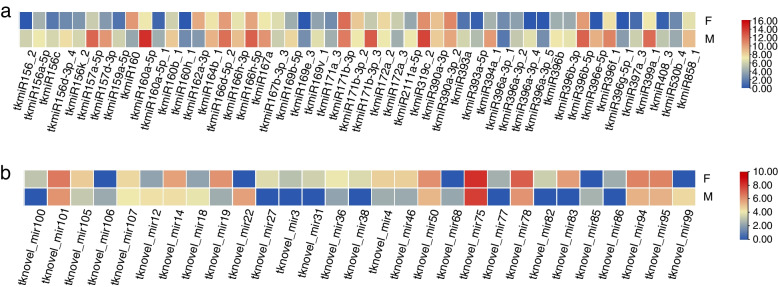
miRNA clustering analysis. **a** known miRNAs clustering; **b** novel miRNAs clustering

To investigate the miRNAs associated with sex differentiation in *TK*, differential expression analysis was performed on the identified miRNAs. As shown in Fig. 2, there were 80 DESs between female and male plants (48 upregulated and 32 downregulated in female plants), including 51 known miRNAs and 29 novel miRNAs. Among the known miRNAs, tkmiR162a-3p, tkmiR160, tkmiR156_2, tkmiR396a-3p_4*,* and tkmiR396e-5p were significantly differentially expressed. Among the novel miRNAs, tknovel_miR83, tknovel_miR27, tknovel_miR38, tknovel_miR31, tknovel_miR82, tknovel_miR100, tknovel_miR3, tknovel_miR77, and tknovel_miR86 were expressed almost exclusively in female flower buds. Notably, tknovel_miR68, tknovel_miR106, tknovel_miR85, tknovel_miR99, and tknovel_miR22 were only expressed in male flower buds.

To better understand the function of DESs, we predicted the target genes of DESs. A total of 3700 target genes were predicted for the DESs (Additional file 4). Among them, 27 novel miRNAs had 282 predicted target genes and 51 known miRNAs had 3418 predicted target genes. The predicted target genes of the same miRNAs ranged from 1 to 278. In addition, many of these target genes are transcription factors, which are closely related to sex differentiation, such as nuclear transcription factor Y subunit A targeted by *MIR169* family members, auxin response factor and floral homeotic gene *APETALA2* targeted by tkmiR160, growth-regulating factor targeted by tkmiR396, AP2-like ethylene-responsive transcription factor targeted by tknovel_miR68 and tkmiR172, squamosa promoter-binding-like gene and SOC1-like MADS-box gene targeted by tkmiR156.

### Validation of miRNA target genes

**Fig. 3:**
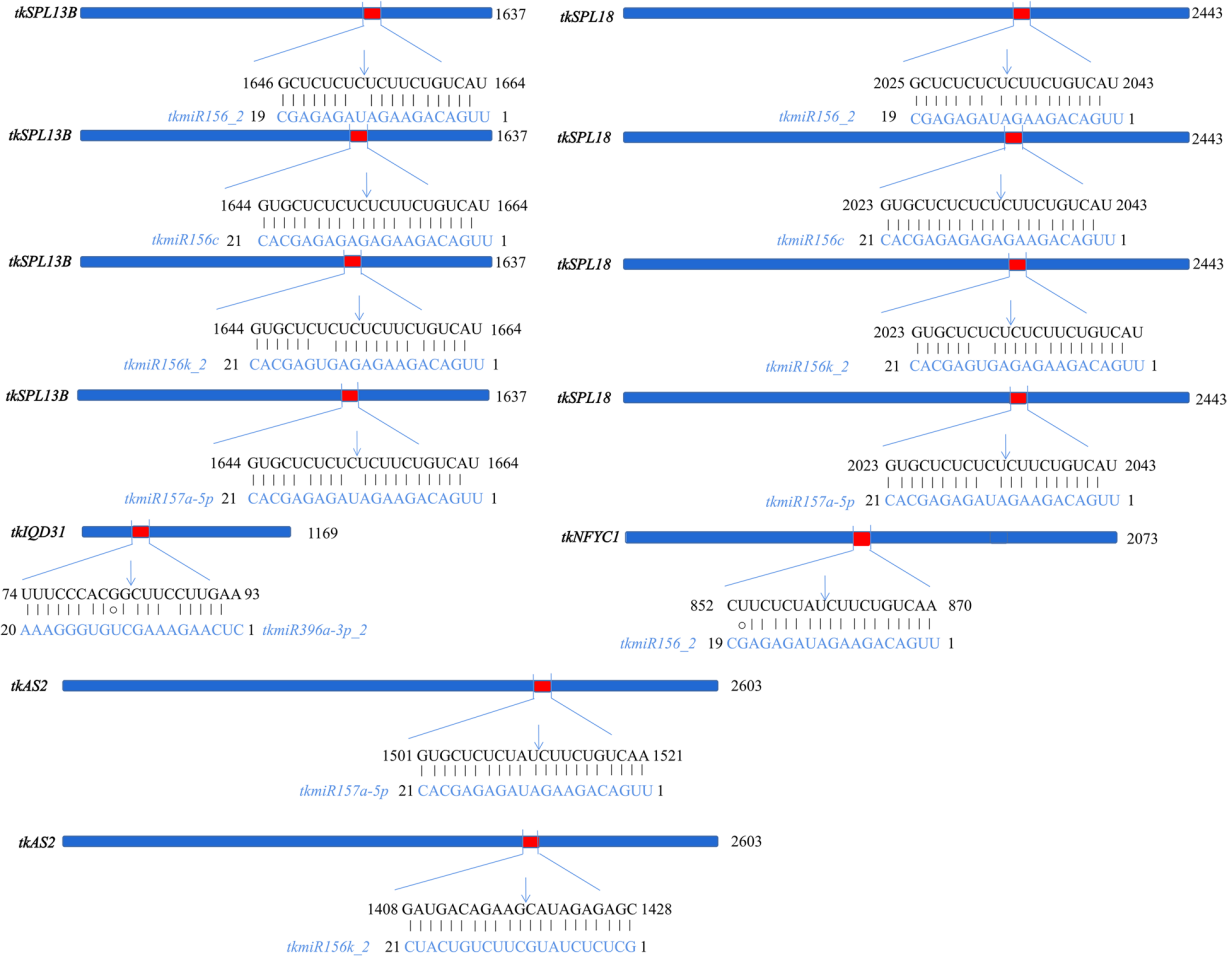
Validation of predicted miRNA targets. The blue nucleotide sequence represents the miRNA, and the black nucleotide sequence represents the corresponding mRNA. Vertical arrows indicate cleavage locations in the target mRNA. Watson-Crick pairing (ǀ) and G:U wobble pairing (○) are indicated

To verify the accuracy of target gene predictions, we randomly validated 6 miRNAs using the modified 5’RACE method. miRNA generally mediates a single cleavage site, and it is generally between the 10th base and the 11th base on it. The experimental results showed that *tkSPL13B* and *tkSPL18* were common target genes for tkmiR156_2, tkmiR156c, tkmiR156k_2, and tkmiR157a-5p. This was consistent with the results of previous studies [33]. In addition, we demonstrated that tkmiR157a-5p and tkmiR156k_2 had a cleavage effect on *tkAS2*, tkmiR156_2 had a cleavage effect on *tkNFYC1*, and *tkIQD31* was the target gene of tkmiR396a-3p_2 (Fig. 3).

### RT-qPCR validation of gene expression patterns

**Fig. 4:**
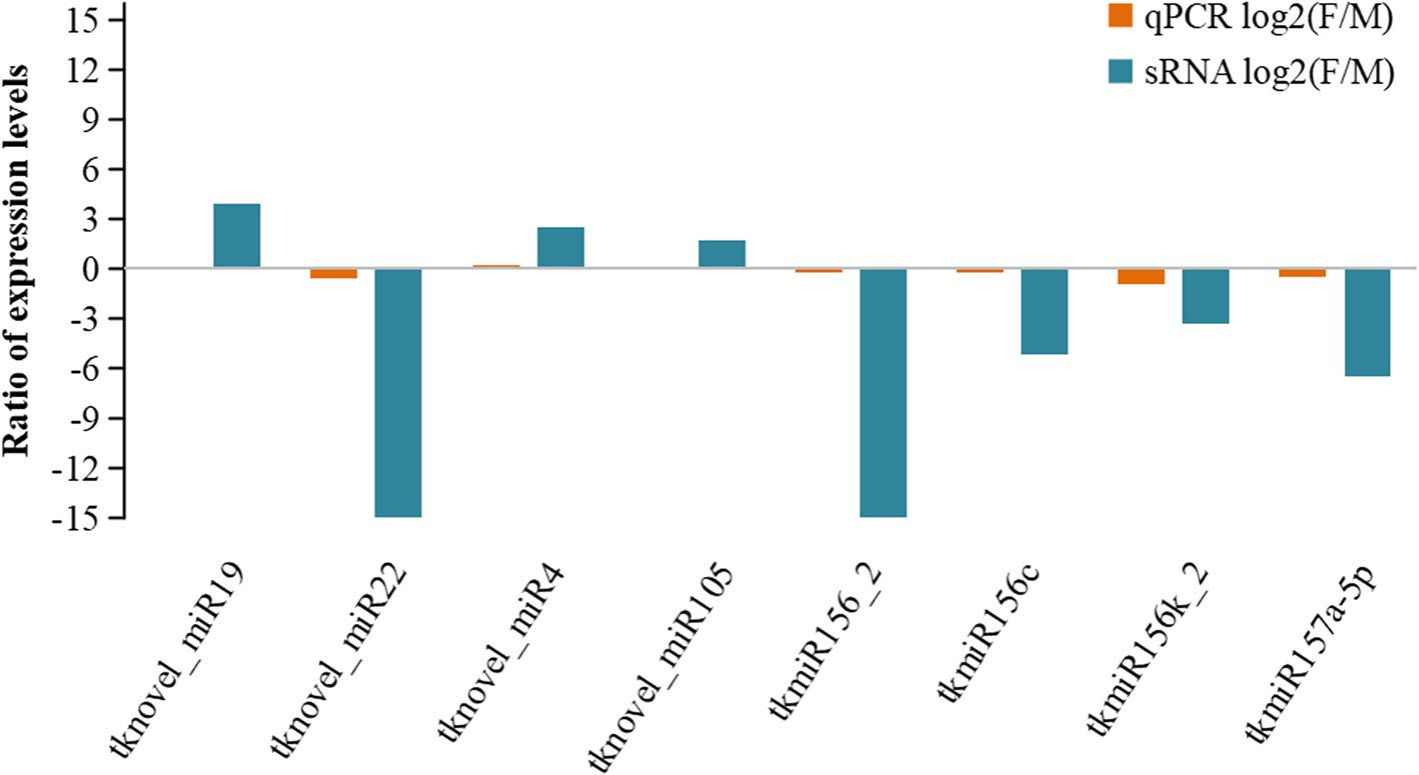
Validation and comparison of sRNA sequencing results by RT-qPCR

To further confirm the sRNA sequencing results, 8 DESs (tknovel_miR19, tknovel_miR22, tknovel_miR4, tknovel_miR105, tkmiR156_2, tkmiR156c, tkmiR156k_2 and tkmiR157a-5p) were validated using RT-qPCR. The expression trend of selected miRNAs were consistent with the sequencing results, indicating that the sRNA sequencing results were reliable (Fig. 4).

### Combined analysis of transcriptome and sRNA sequencing

**Fig. 5:**
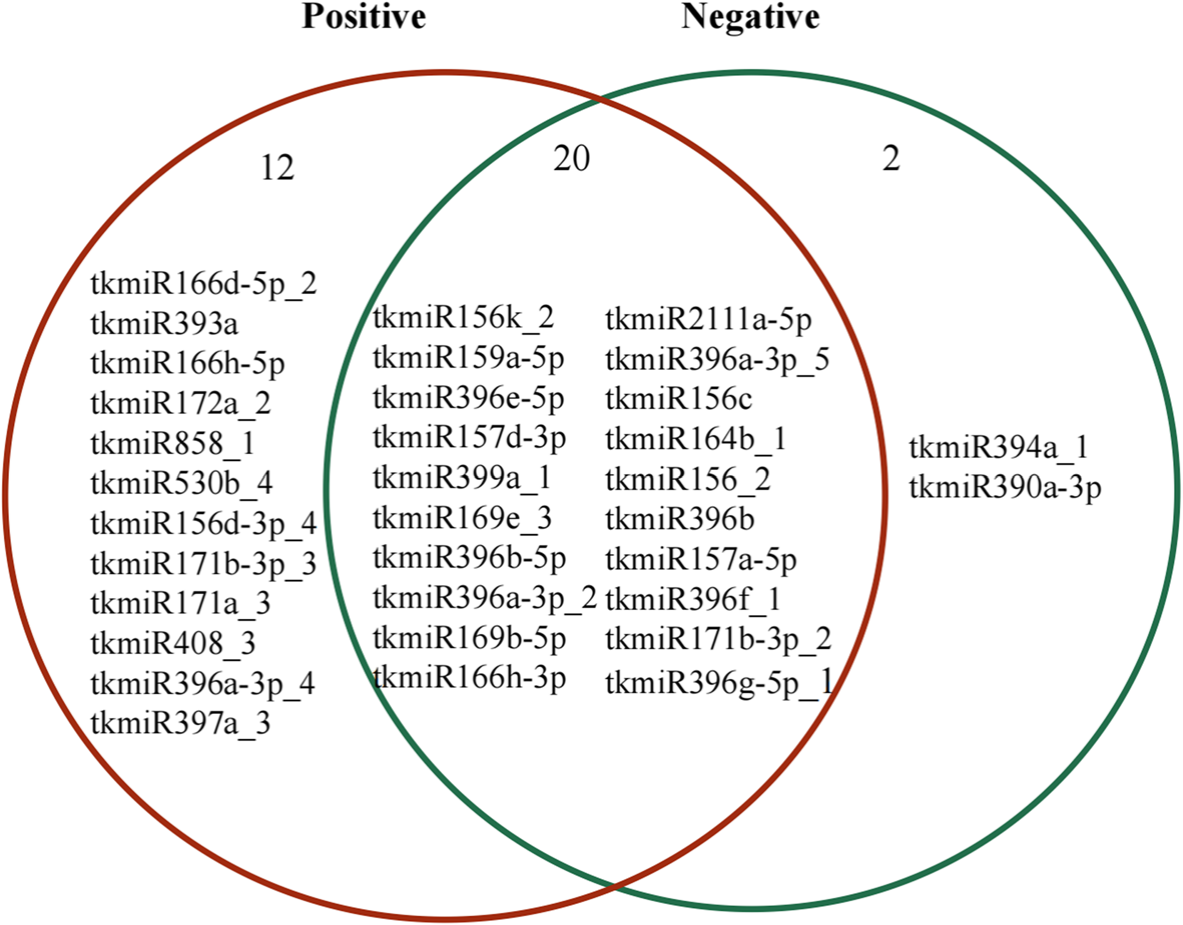
Positive and negative regulated miRNAs in F vs. M

The results of the multi-omics analysis show that miRNAs with negative regulatory effects on target genes totaled 22 and were annotated to 107 target genes; positive regulators totaled 32 and were annotated to 124 target genes; and there are 20 miRNAs with both positive and negative regulatory roles (Fig. 5; Additional file 5).

**Fig. 6:**
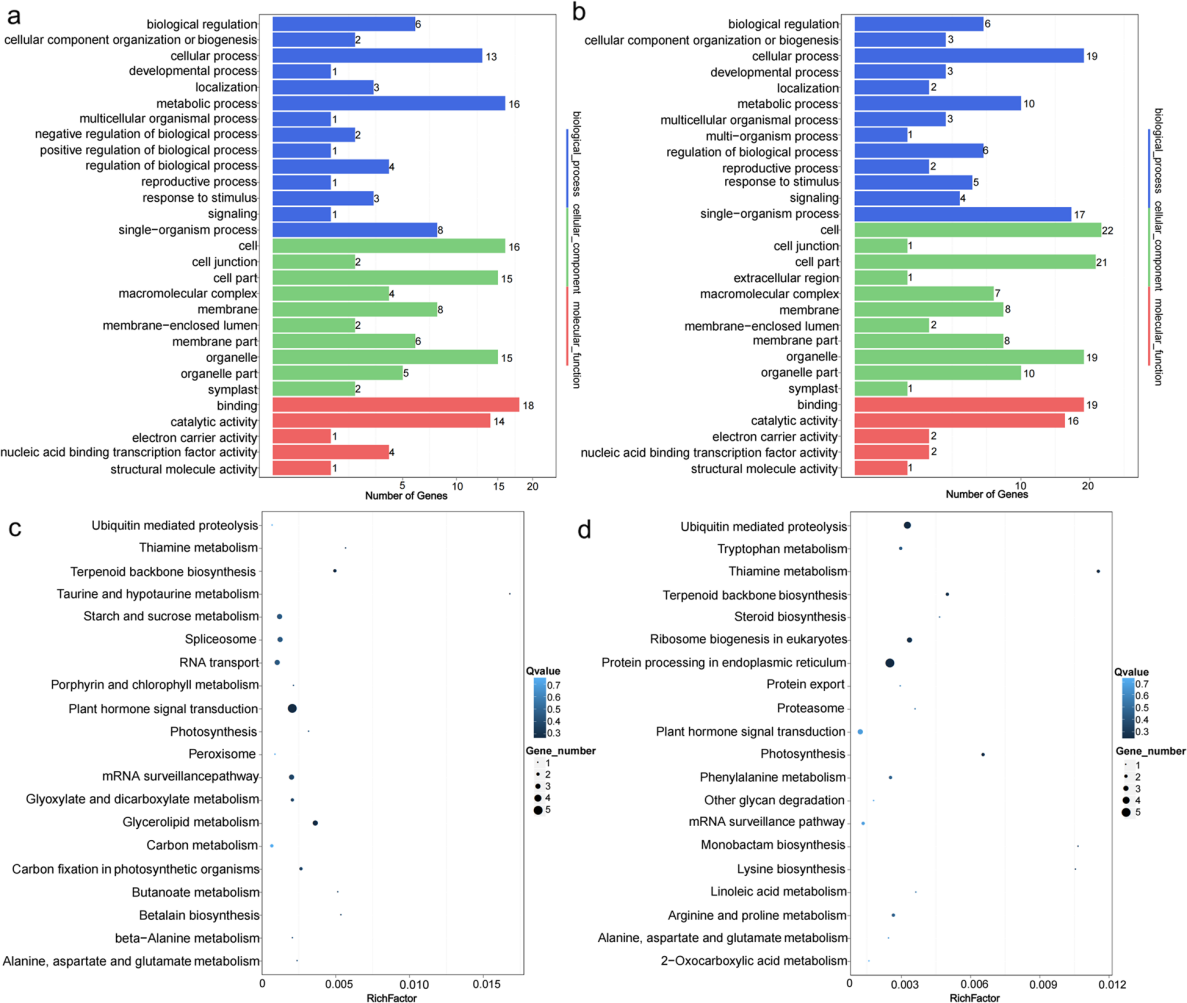
GO functional classification and KEGG pathway annotation classification of target genes. **a** GO functional classification of negatively correlated target genes; **b** GO functional classification of positively correlated target genes; **c** Negatively correlated target gene pathway annotation; **d** Positively correlated target gene pathway annotation. The size and color of the bubble represent the number of enriched genes and enrichment significance, respectively. KEGG analysis was performed based on the method as described by Kanehisa et al. [34]

GO and KEGG enrichment analyses were conducted on positively and negatively correlated target genes. All target genes were annotated to 315 GO terms (Additional file 6). These GO terms were assigned to three categories: biological process (BP), cellular component (CC), and molecular function (MF). The negatively correlated target genes were involved in 175 GO terms (Fig. 6a). In the BP category, the most enriched GO term was a metabolic process. In the CC category, the most enriched GO term was cells. In the MF category, the most enriched GO term was binding. The 88 positively associated target genes were involved in 221 GO terms (Fig. 6b). In the BP category, the most enriched GO term was a cellular process. In the CC category, the most enriched GO term was the cell. In the MF category, the most enriched GO term was binding. It is worth noting that there are 2 target genes (CL12610.Contig2_All, CL12610.Contig1_All) enriched in the process of flower organ development (GO:0048437), floral whorl development (GO:0048438) and flower development (GO:0009908) at the same time, indicating that its corresponding miRNAs may play an important role in the process of sex differentiation of *TK*.

The biological characteristics of target genes were investigated by KEGG pathway enrichment analysis. The top 20 KEGG pathways with Q < 0.05 are shown in Fig. 6c and d. KEGG metabolic pathway analysis showed that negatively associated target genes were mainly enriched in plant hormone signal transduction, while positively associated target genes were mainly enriched in protein processing in the endoplasmic reticulum.

### Screening of core miRNAs and target genes

**Fig. 7:**
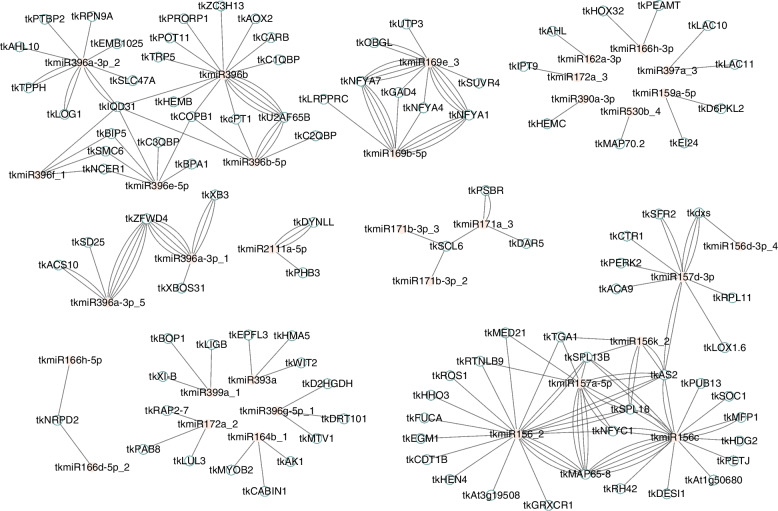
Network regulation map of miRNAs and their target genes. The network includes 133 nodes and 201 edges. The pink ellipses represent the miRNAs and the blue ellipses represent the target genes

Using Cytoscape software, we constructed a regulatory network map between DESs and target genes (Fig. 7). On this basis, using the cytohubba plugin in Cytoscape software, we found the top 12 core genes in the regulatory network. As shown in Table 2, these core miRNAs are in turn tkmiR156c, tkmiR156_2, tkmiR157a-5p, tkmiR157d-3p, tkmiR396b, tkmiR396a-3p_2 and tkmiR156k_2. And core target genes include *tkSPL18*, *tkSPL13B*, *tkAS2*, *tkIQD31*, and *tkNFC1*.

**Table 2:** Top 12 core genes in regulatory network of miRNA and target genes

Rank	Name	Score
1	tkmiR156c	2146
2	tkmiR156_2	2130
3	*tkSPL18*	1144
4	*tkSPL13B*	872
5	*tkAS2*	778
6	tkmiR157a-5p	516
7	tkmiR157d-3p	440
8	tkmiR396b	394
9	*tkIQD31*	288
10	tkmiR396a-3p_2	280
11	*tkNFYC1*	226
12	tkmiR156k_2	164

**Fig. 8:**
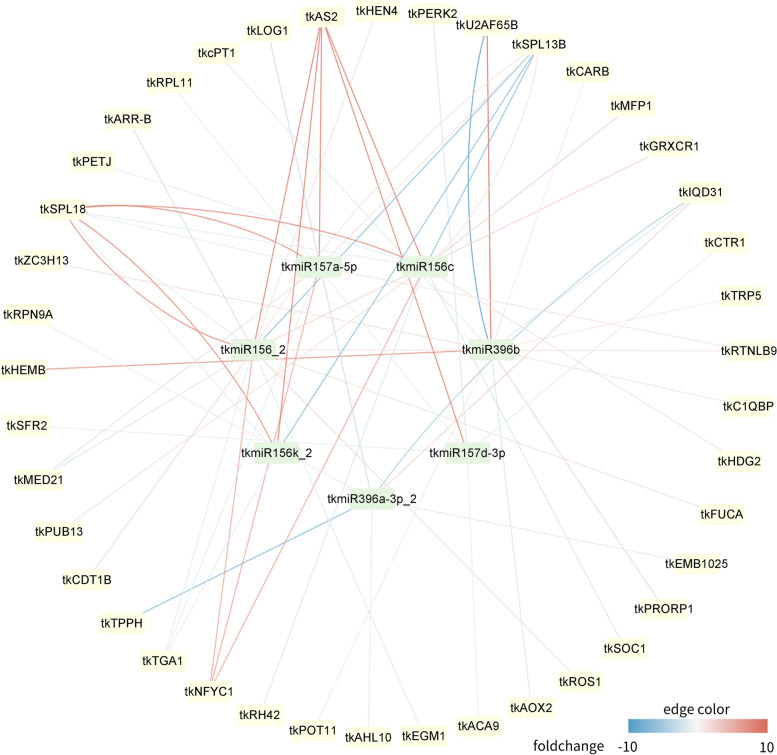
Regulatory network map of candidate miRNAs and target genes. The network includes 48 nodes and 66 edges. The green rectangles represent the miRNAs and the yellow ellipses represent the target genes. The color of the edge represents the degree of regulation of the target genes by the miRNAs

Around the above miRNAs, we constructed the interaction network map of these miRNAs and target genes (Fig. 8). In addition to the key target genes mentioned above, the map also shows all target genes that have positive and negative regulatory relationships with miRNAs. Some target genes are only positively regulated by miRNAs, such as *tkHEMB*, *tkFUCA*, and *tkHDG2*; and some are only negatively regulated, such as *tkLOG1*, *SOC1*, and *cPT1*. At the same time, there are also some target genes and miRNAs whose regulatory relationship is complex, both positively and negatively regulated, such as *tkAS2*, *tkSPL18*, *tkSPL13B*, *tkU2AF65B.*

**Fig. 9:**
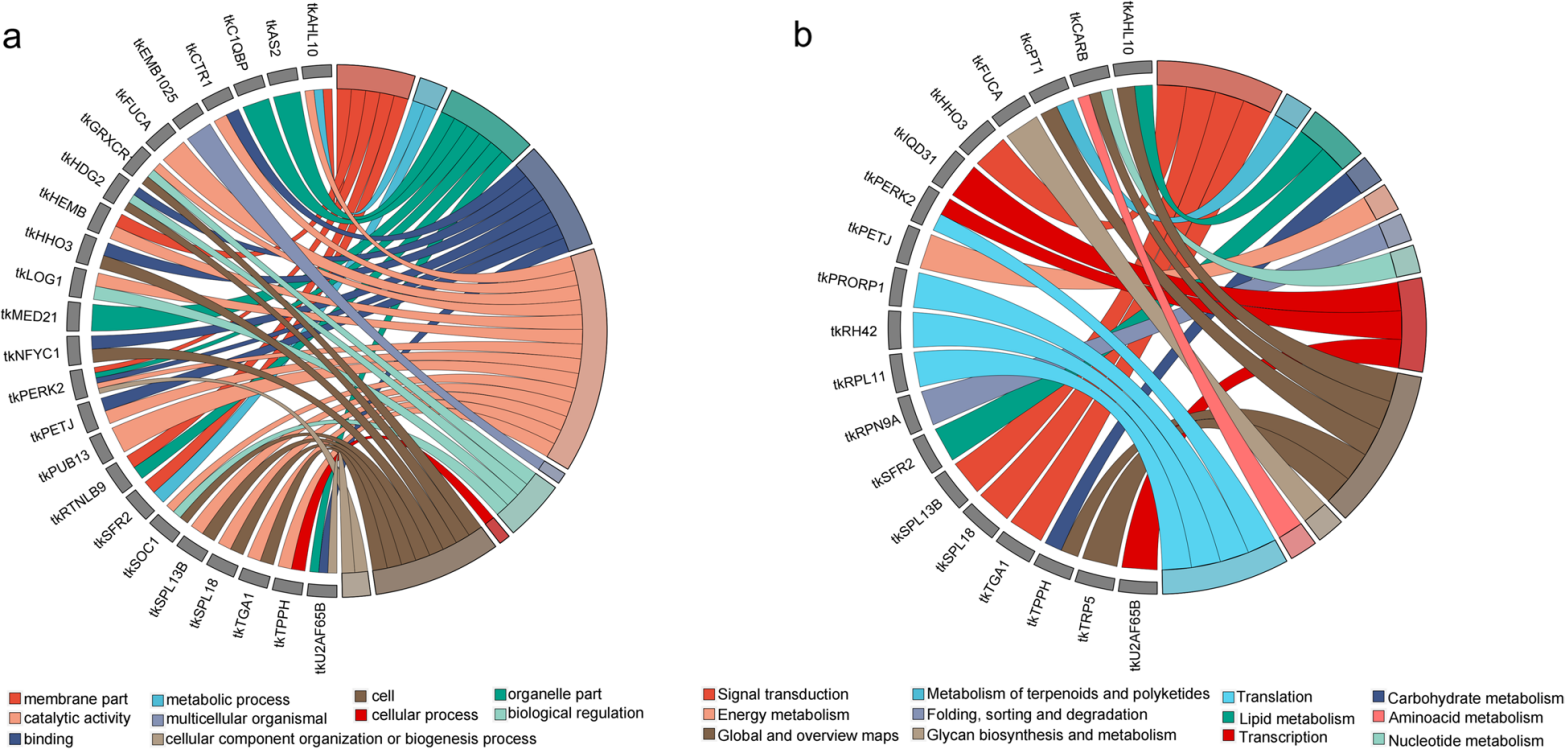
Target genes functional annotation of candidate miRNAs. **a** GO enrichment analysis; **b** KEGG pathway analysis

To better study the role of these target genes in the sex differentiation of *TK*, GO and KEGG enrichment analysis was performed on them. Although there are 41 target genes of candidate miRNAs, there are cases that cannot be annotated when performing GO and KEGG enrichment analysis. In the GO analysis, 24 target genes were annotated, and most of them were involved in catalytic activities, binding and cell activities (Fig. 9a); in the KEGG pathway analysis, 19 target genes were annotated, including genes involved in global and overview maps, translation, and transcription (Fig. 9b).

## Discussion

The determination and differentiation of sex in plants have become the focus of developmental genetics research in recent years [35–37]. Compared with animals, plants have more diverse sex determination patterns. Studies have shown that flowers are not only influenced by the external environment, but also by internal genes. Stamens and pistils require a large number of specific genes to participate in each developmental stage, among which miRNAs play an important role in the gene regulation process.

In this study, sRNA libraries of female and male flower buds of *TK* were constructed. All samples had a Q20 score of more than 99%, indicating high-quality sequencing data. The analysis results showed that there were obvious differences in the miRNA of male and female flower buds. Among them, the average raw tag of female flower buds was 26,458,609, while the male flower buds were 25,535,046. The statistical results of miRNA length distribution showed that the number of miRNAs in male flower buds was higher than that of female flower buds between 18 and 21 nt, and the opposite was true between 26 and 30 nt; Between 22 and 25 nt, the number of miRNAs is comparable. An average of 89 known miRNAs and 34 novel miRNAs were detected in three female flower bud samples, while male flower buds detected an average of 104 known miRNAs and 31 novel miRNAs.

Liu et al. [38] overexpressed the precursor of *GhmiRNA157* in *Z. mays*, and found that the ovule decreased. They believed that this process was due to *miR157* targeting the regulation of *SPL* gene, and then regulating MADS-box gene and auxin signal transduction to control ovule production. As a member of the *MIR157* family, tkmiR157a-5p targets *tkSPL18* and *tkSPL13B*. The transcriptome sequencing results showed that the expression level of *tkSPL18* (CL104. Contig11_All) in male flower buds (FPKM = 0.46) was much higher than that in female flower buds (FPKM = 0); The expression of *tkSPL13B* (CL104. Contig21_All) in female flower buds was 0.46, but not in male flower buds. KEGG prediction shows that these two genes encode BRASSINOSTEROID-INSENSITIVE 1 (BRI1) and participate in the biosynthesis of brassinosteroid (BR) in the signal transduction process. BRI1 is a leucine-rich repeat receptor-like protein kinase, which is a key component of the transmembrane BR receptor. Previous studies have shown that in *Z. mays*, the interaction of BR and jasmonic acid inhibits the development of the male flowers [39, 40]. Interestingly, they are also affected by tkmiR156c, tkmiR156_2, and tkmiR156k_2. Therefore, it is possible for these four miRNAs to participate in the biosynthesis process of BR by targeting *tkSPL18* and *tkSPL13B*, and then regulating the sex of *TK*. In addition to the four miRNAs, the other three core target genes are also highly likely to participate in the gender differentiation of *TK*. For example, *tkAS2* (CL12610. Contig2_All, CL12610. Contig1_All), the target gene of tkmiR157d-3p, is also subject to tkmiR156c, tkmiR156_2, tkmiR156k_2 and tkmiR157a-5p are positively regulated. In GO, it is considered to participate in the process of floral whorl development, flower organ development, and plant hormone signal transformation. Unfortunately, KEGG pathway enrichment has not been annotated with this gene, and the specific metabolic pathway involved in this gene is unknown. Yang et al. [41] found that *Nicotiana tabacum* overexpressing CemiR396 showed pistil bending and reduced fertility, implying the conserved role of CemiR396 in floral development. Liang et al. [42] showed that miR396 can control the number of carpels and pistil development of *Arabidopsis* by regulating the GRF/GIF complex. In *TK*, tkmiR396b and tkmiR396a-3p_2, whether it can play a similar role remains to be further studied.

## Conclusion

In this study, we identified a total of 80 DESs. To further narrow down gender-related candidate miRNAs, we constructed a regulatory network between target genes and miRNAs, and screened seven core miRNAs: tkmiR156c, tkmiR156_2, tkmiR157a-5p, tkmiR157d-3p, tkmiR396b, tkmiR396a-3p_2 and tkmiR156k_2. They are closely related to the sex differentiation of the *TK*. The identification of these miRNAs broadened our understanding of miRNA regulation of sex in *TK* and provided a theoretical basis for further exploration of the sex regulation mechanism of *TK*.

## Supplementary Information

**Additional file 1.** The primers for 5'RACE and RT-qPCR.

**Additional file 2.** The stem-loop structures for miRNA.

**Additional file 3.** The mature and precursor sequences for identified miRNAs.

**Additional file 4.** F-vs-M DESs and predicted targets.

**Additional file 5.** F-vs-M positive and negative miRNAs.

**Additional file 6.** GO terms enriched for target genes.

## Data Availability

The raw sequence data reported in this paper have been deposited in the Genome Sequence Archive (Genomics, Proteomics & Bioinformatics 2021) at National Genomics Data Center (Nucleic Acids Res 2022), China National Center for Bioinformation / Beijing Institute of Genomics, Chinese Academy of Sciences (GSA: CRA008425) that are publicly accessible at https://ngdc.cncb.ac.cn/gsa.

## Abbreviations

*TK*: *Trichosanthes kirilowii* Maxim.
DESs: Differentially expressed miRNAs
F: Female samples
M: Male samples
