# Supplementary material for: Identification and analysis of miRNAs differentially expressed in male and female Trichosanthes kirilowii maxim
Source: BMC Genomics. 2023 Feb 21;24:81. doi: 10.1186/s12864-023-09178-8 (PMC9945594; doi:10.1186/s12864-023-09178-8)

Additional file 2. The stem-loop structures for miRNA

tknovel_miR5 MFE= -40.40 kcal/mol

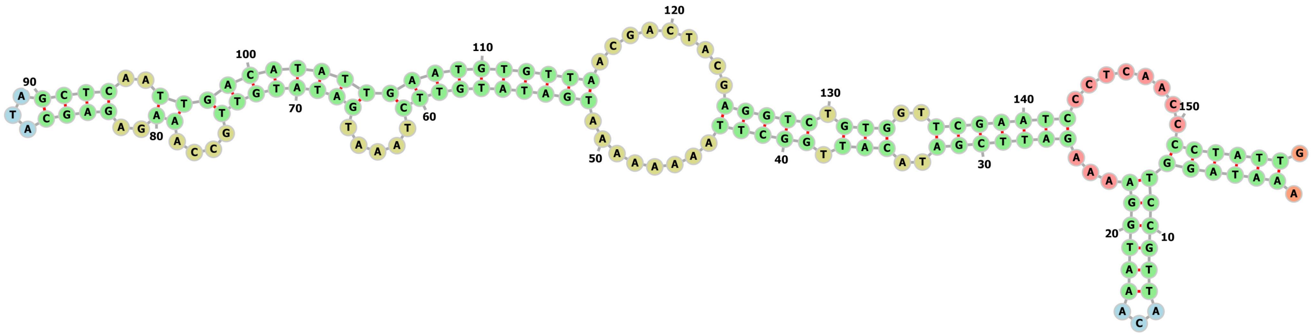

tknovel_miR7 MFE= -29.10 kcal/mol

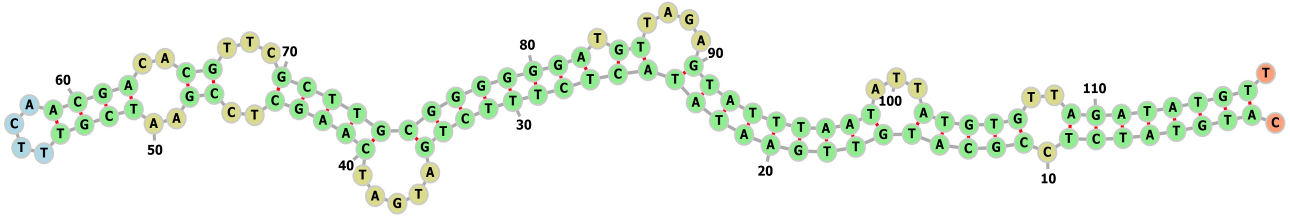

tknovel_miR9 MFE= -86.50 kcal/mol

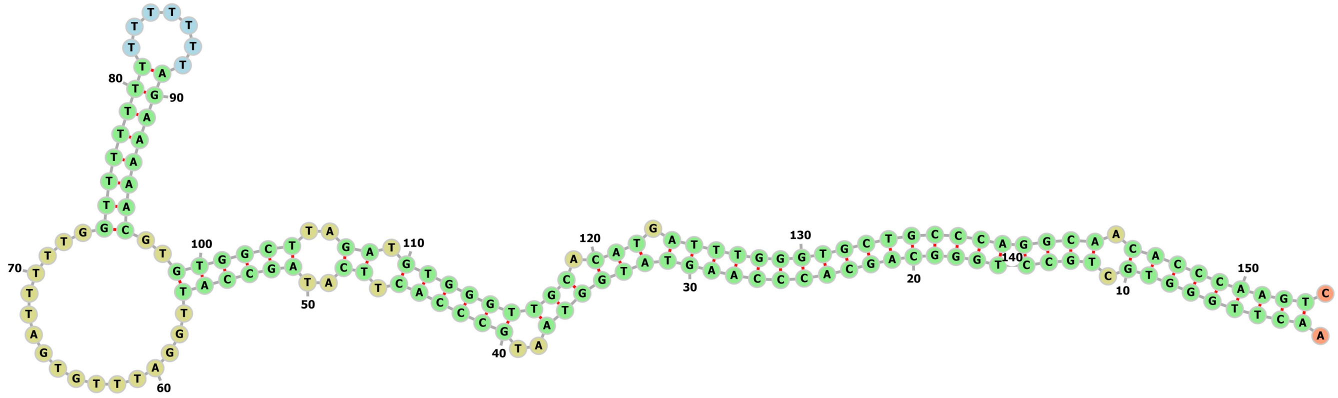

tknovel_miR11 MFE= -58.80 kcal/mol

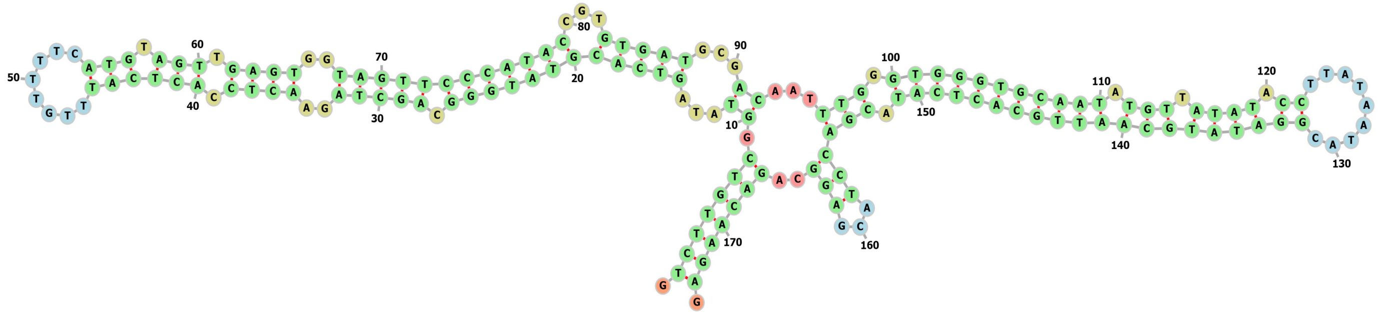

tknovel_miR13 MFE= -24.00 kcal/mol

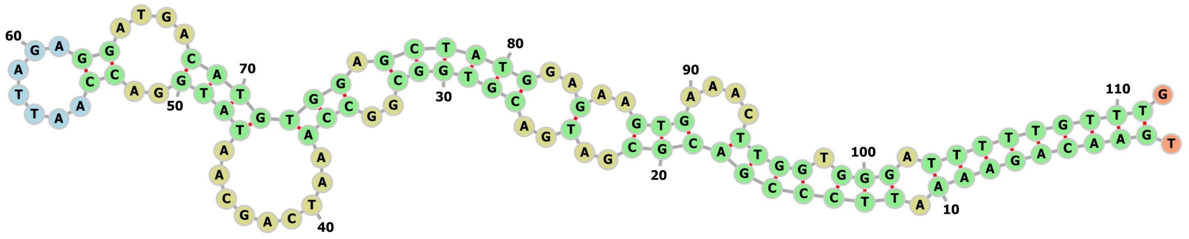

tknovel_miR17 MFE= -46.00 kcal/mol

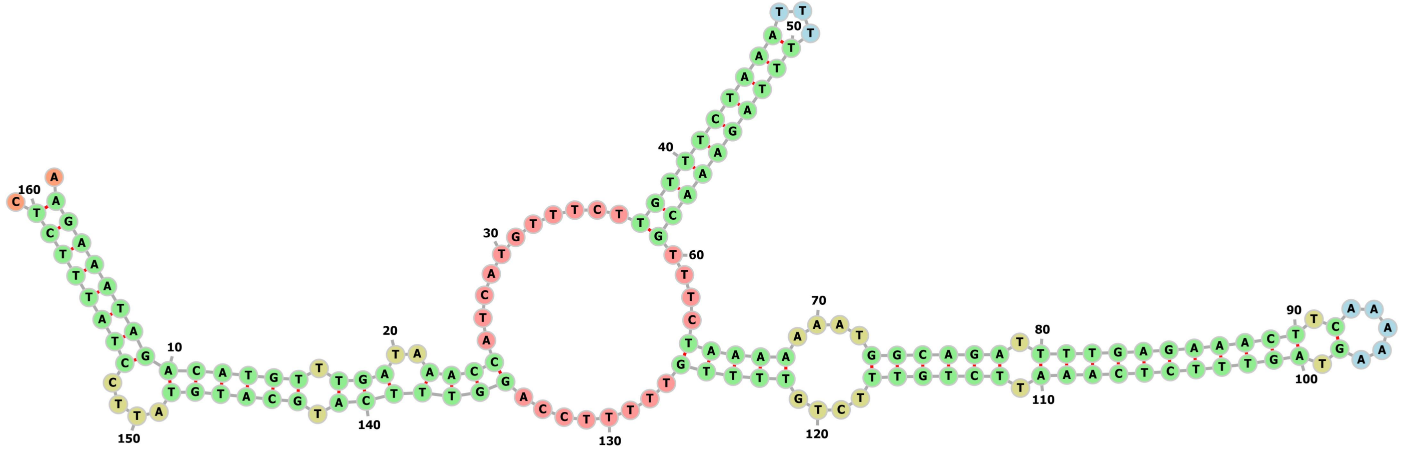

tknovel_miR18 MFE= -44.80 kcal/mol

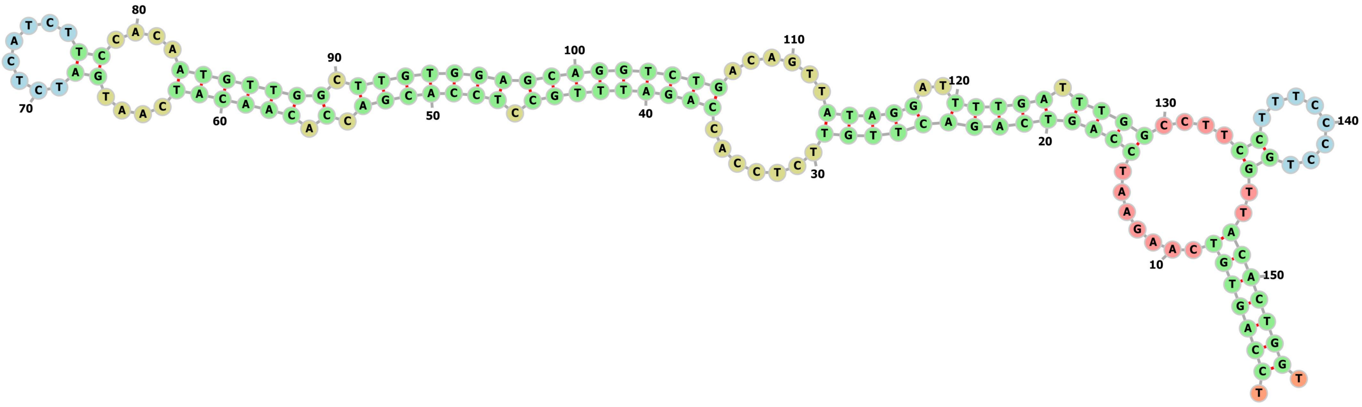

tknovel_miR21 MFE= -37.40 kcal/mol

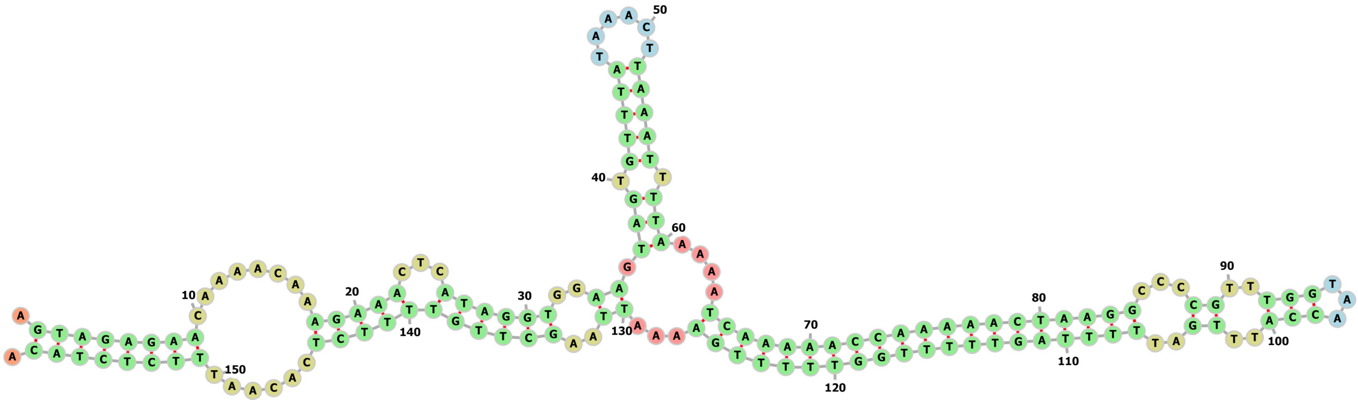

tknovel_miR25 MFE= -31.70 kcal/mol

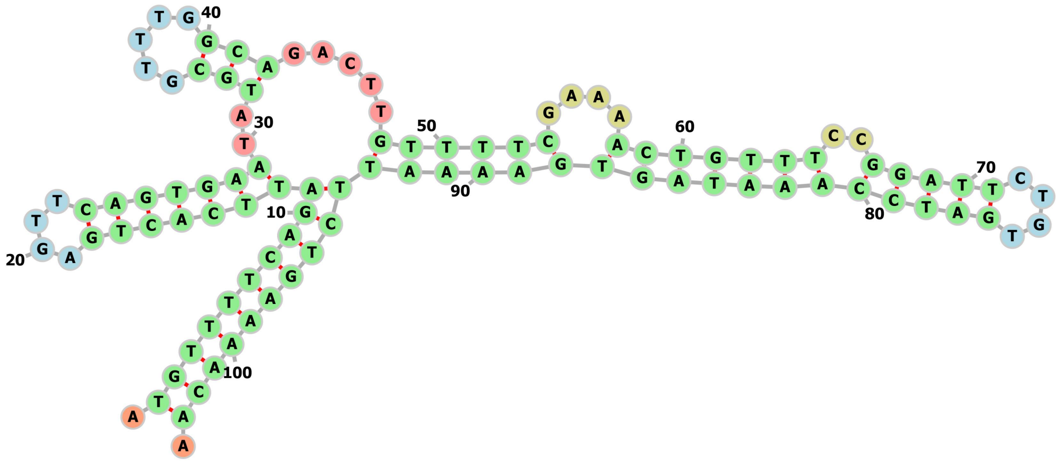

tknovel_miR30 MFE= -24.40 kcal/mol

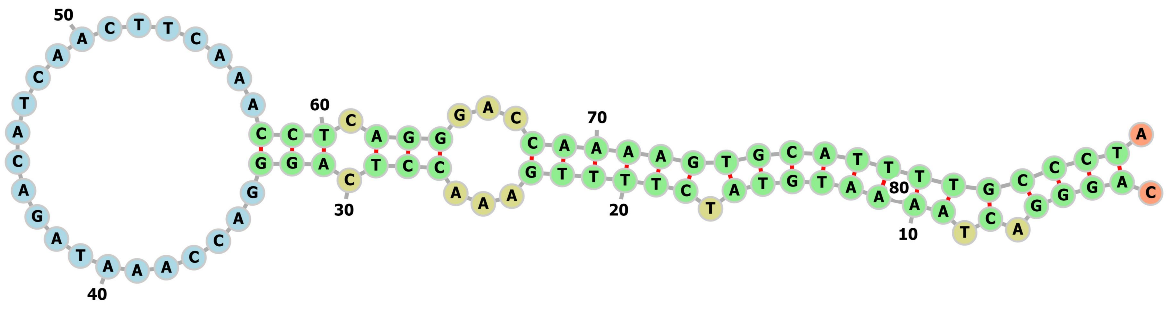

tknovel_miR31 MFE= -80.60 kcal/mol

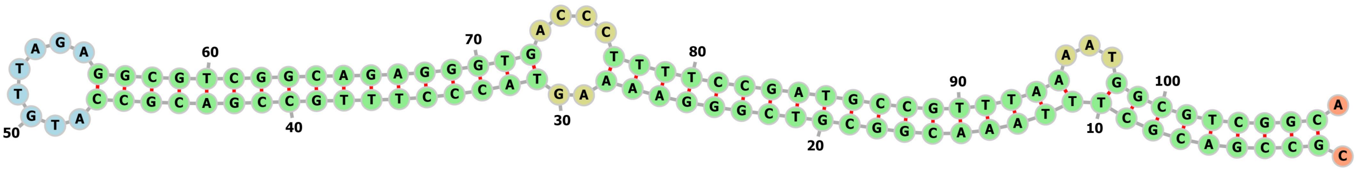

tknovel_miR33 MFE= -43.80 kcal/mol

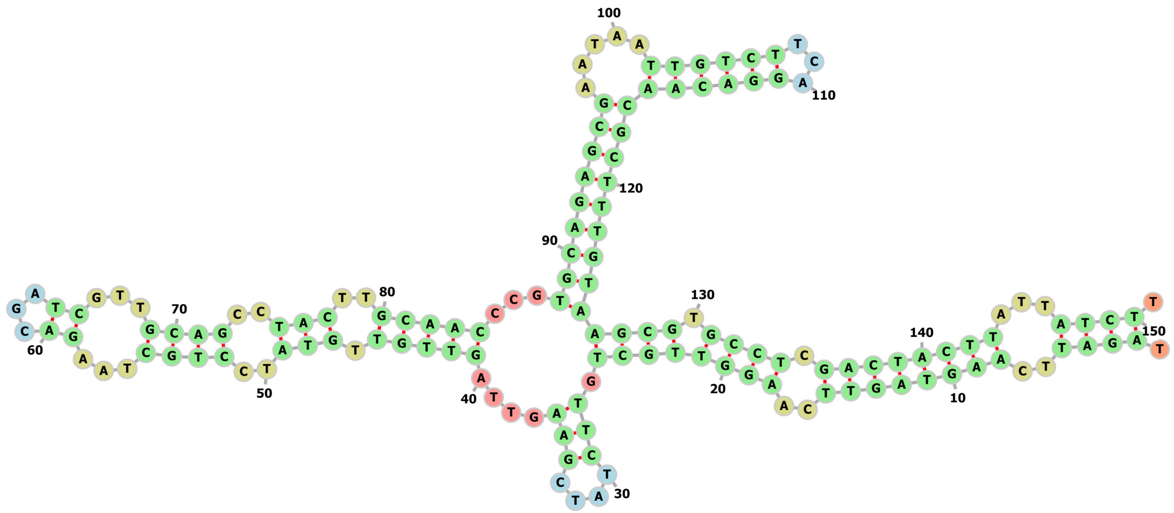

tknovel_miR40 MFE= -53.80 kcal/mol

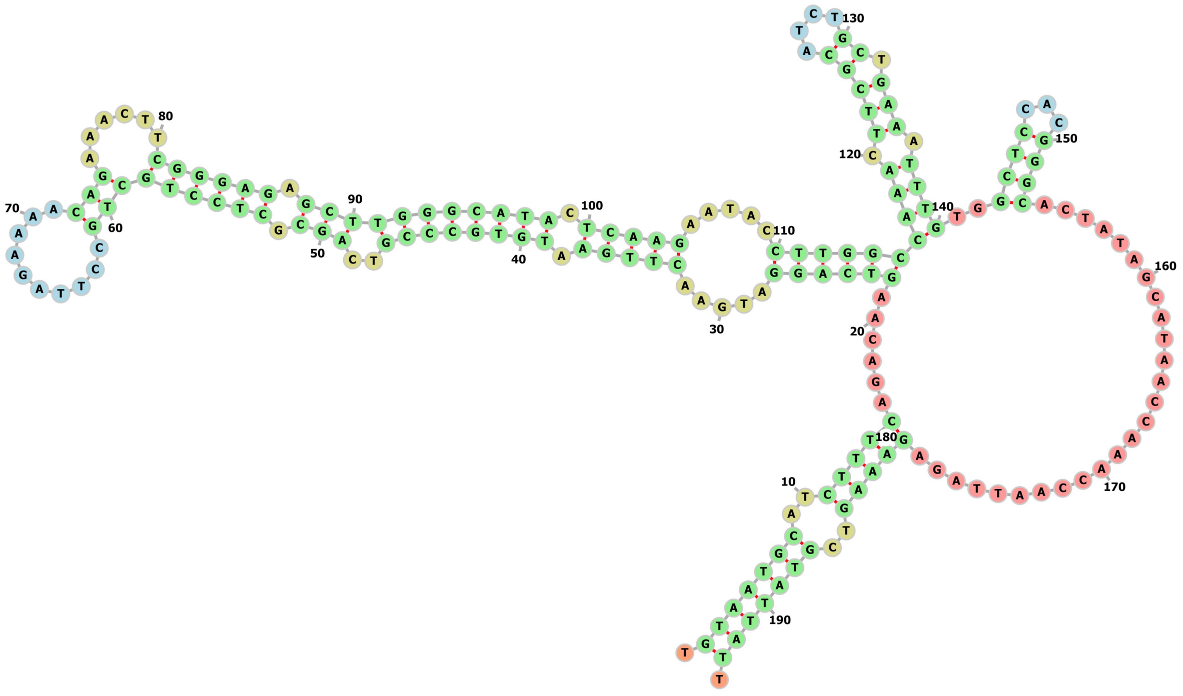

tknovel_miR41 MFE= -48.50 kcal/mol

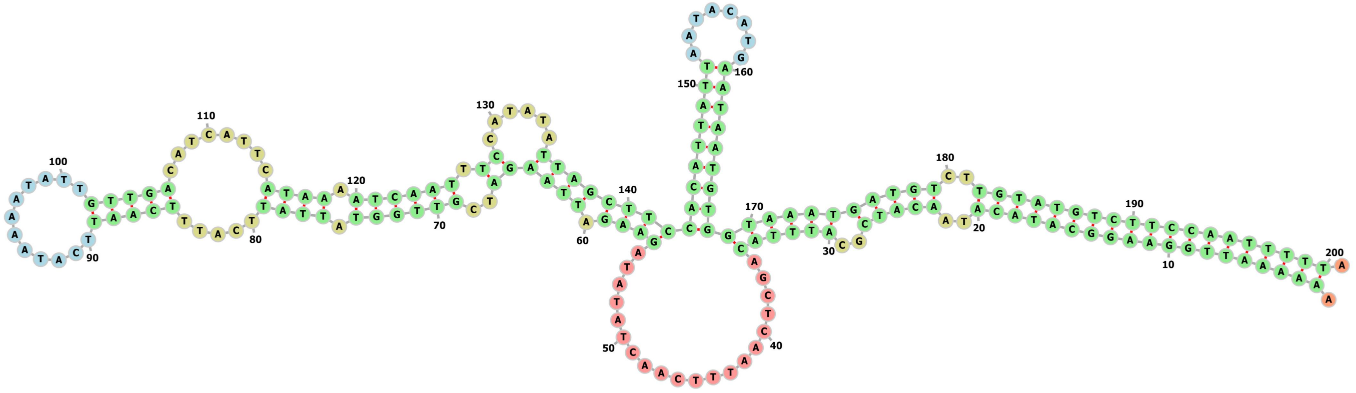

tknovel_miR42 MFE= -53.50 kcal/mol

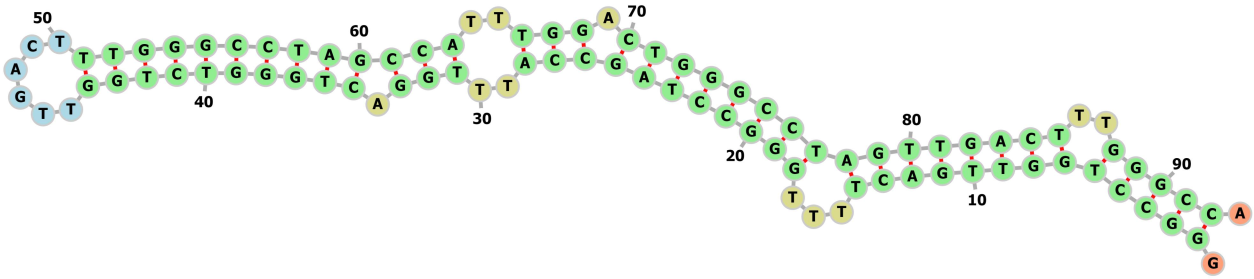

tknovel_miR43 MFE= -42.70 kcal/mol

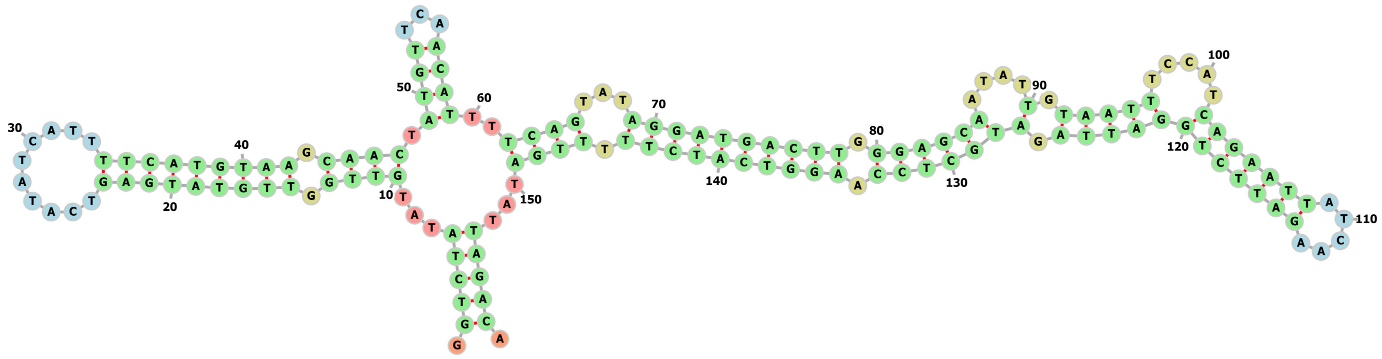

tknovel_miR45 MFE= -63.70 kcal/mol

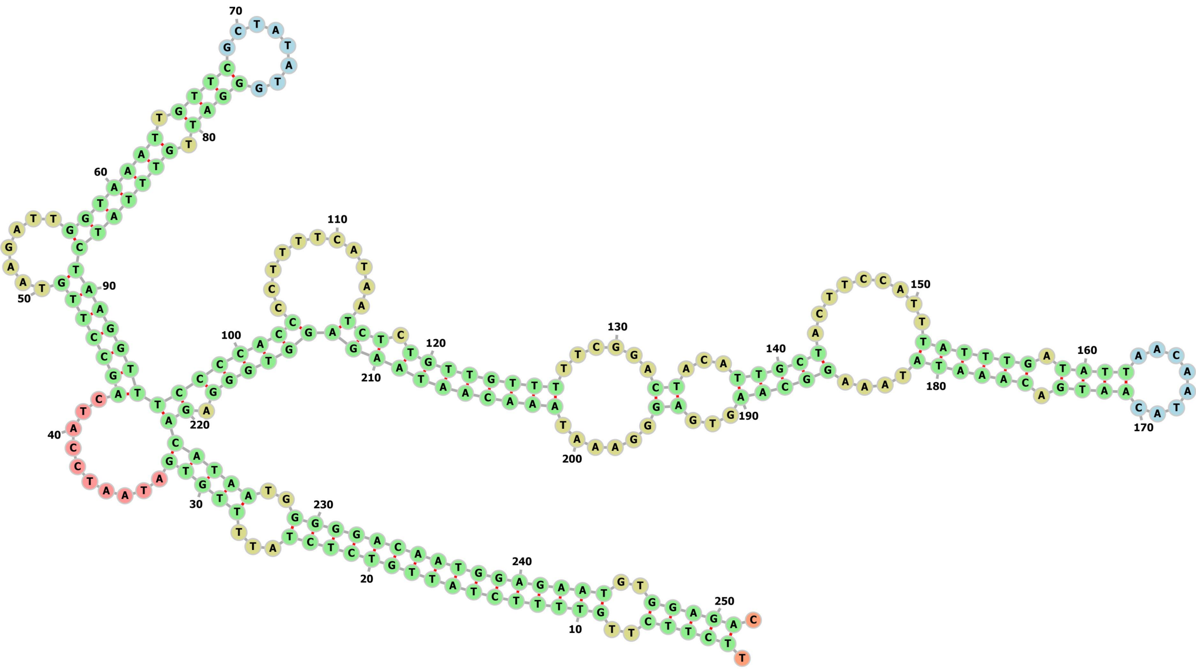

tknovel_miR46 MFE= -24.27 kcal/mol

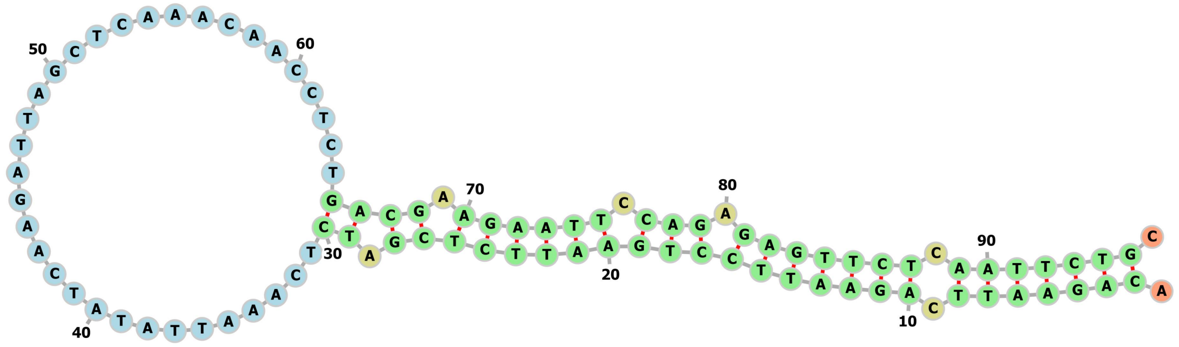

tknovel_miR47 MFE= -97.40 kcal/mol

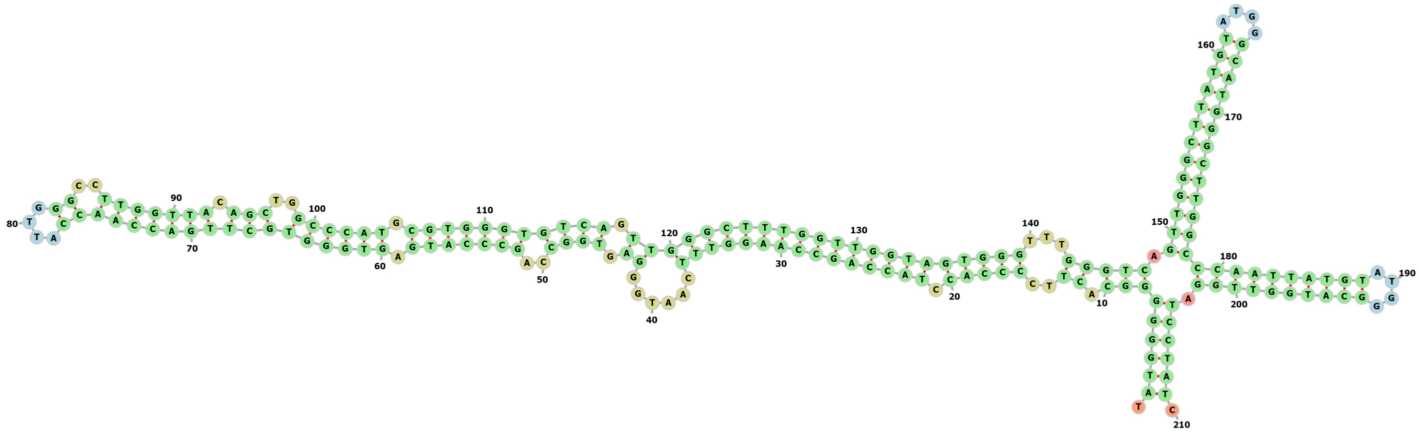

tknovel_miR48 MFE= -28.90 kcal/mol

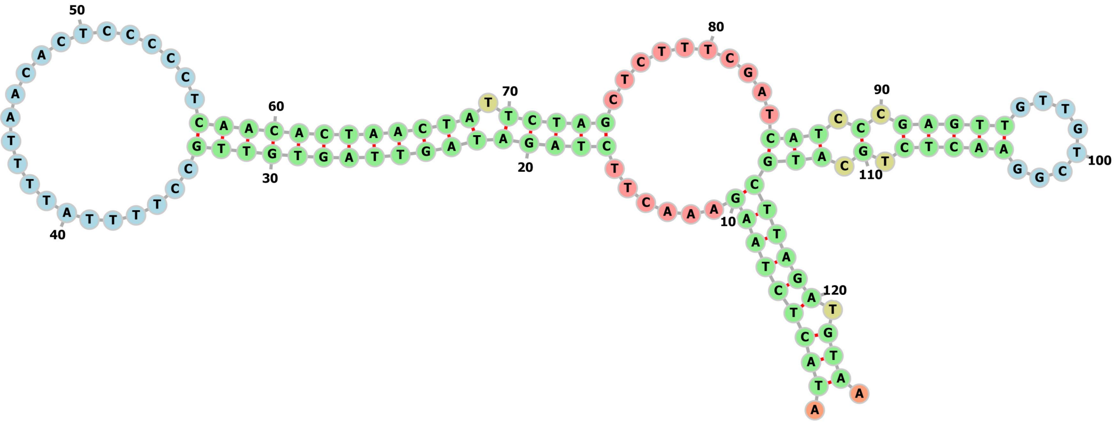

tknovel_miR50 MFE= -37.00 kcal/mol

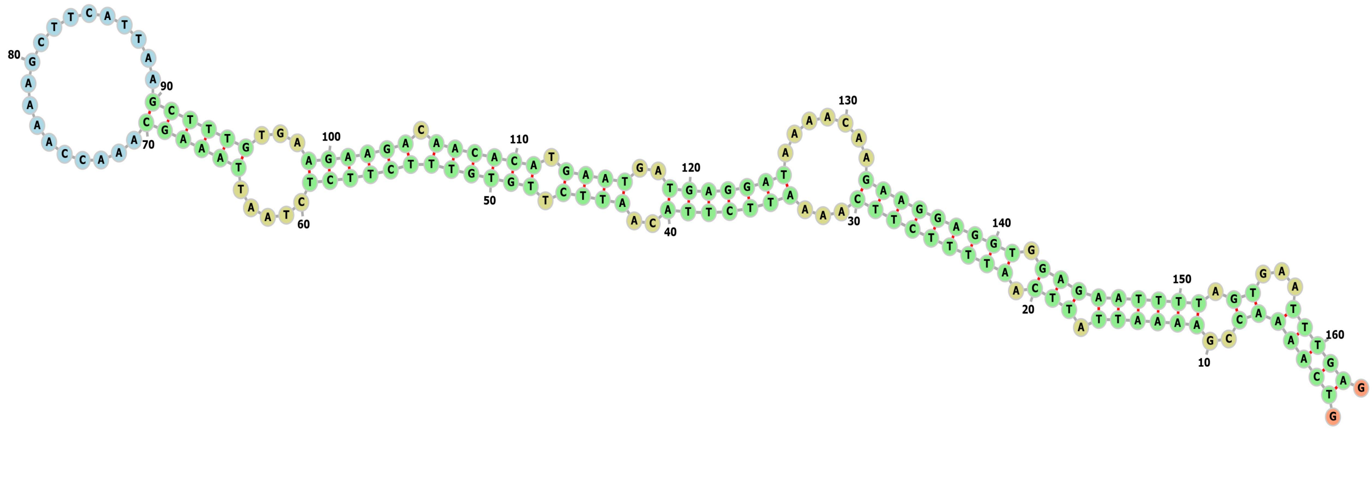

tknovel_miR51 MFE= -105.40 kcal/mol

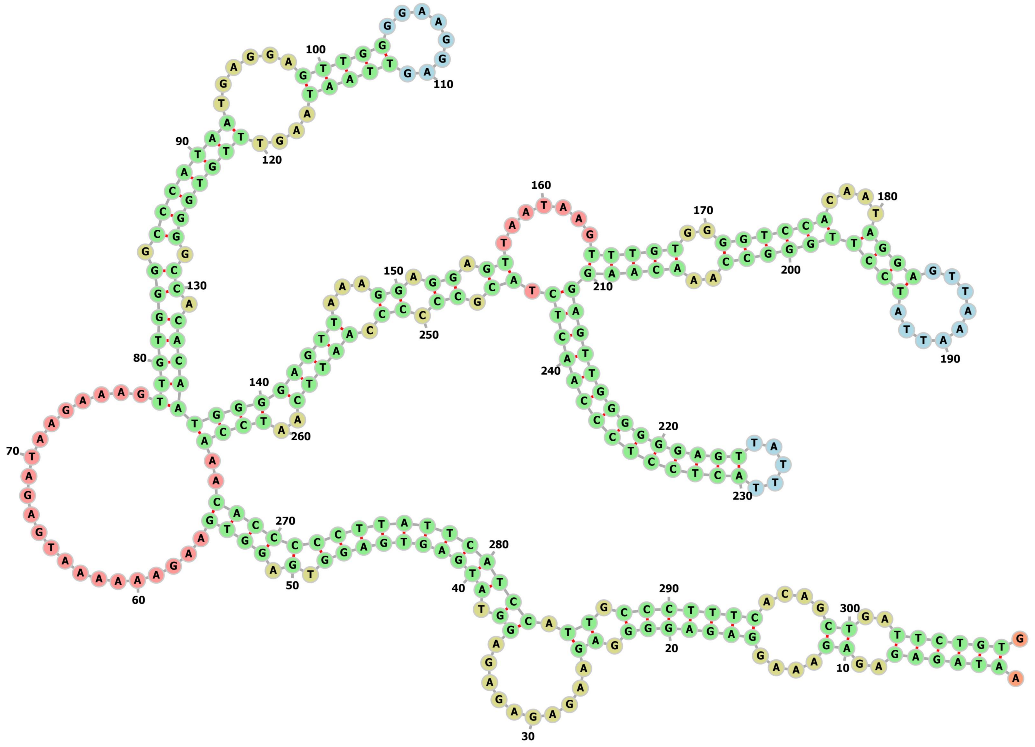

tknovel_miR52 MFE= -52.10 kcal/mol

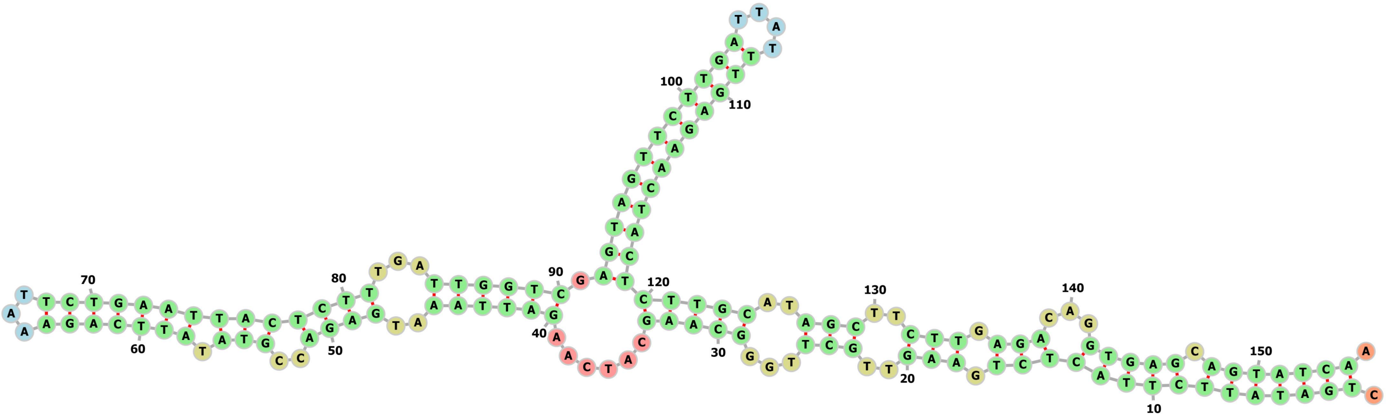

tknovel_miR53 MFE= -44.50 kcal/mol

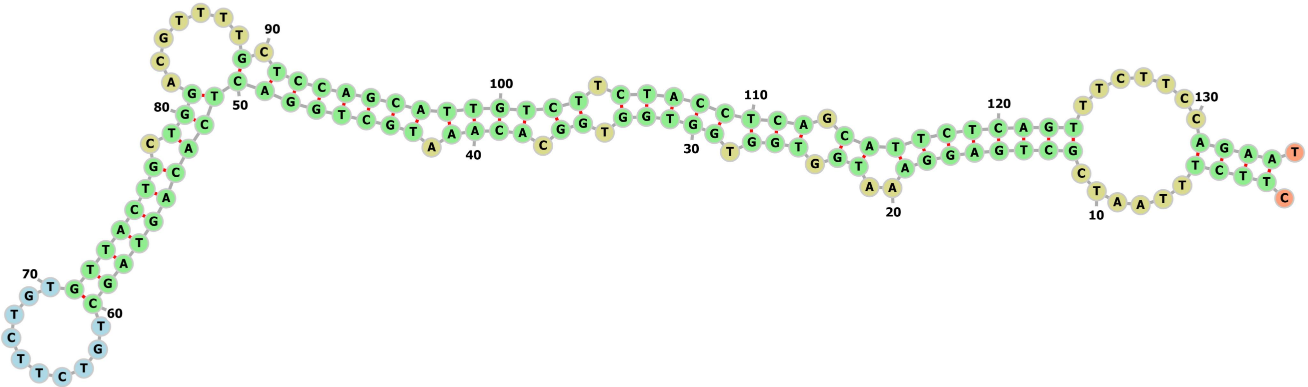

tknovel_miR54 MFE= -57.40 kcal/mol

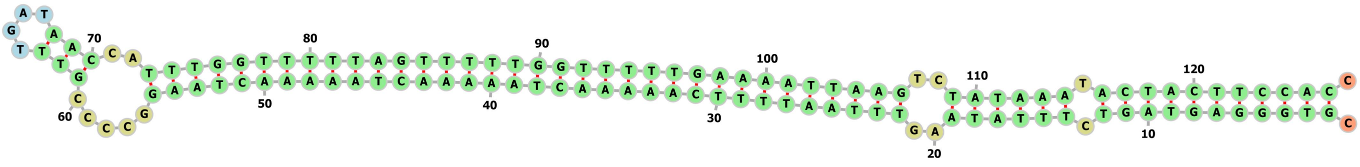

tknovel_miR55 MFE= -47.80 kcal/mol

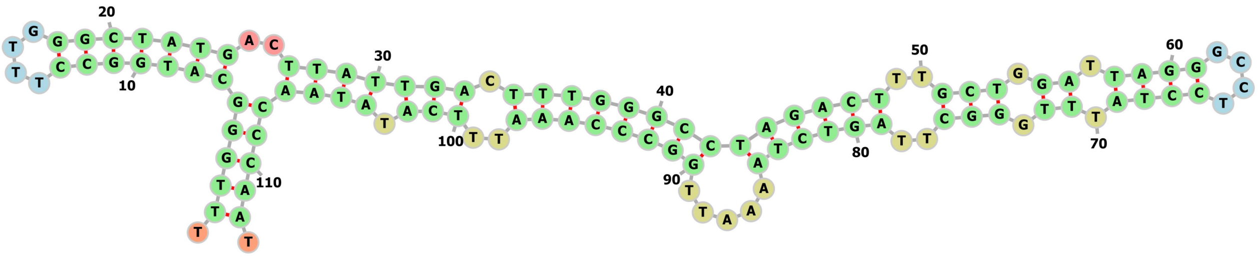

tknovel_miR56 MFE= -41.20 kcal/mol

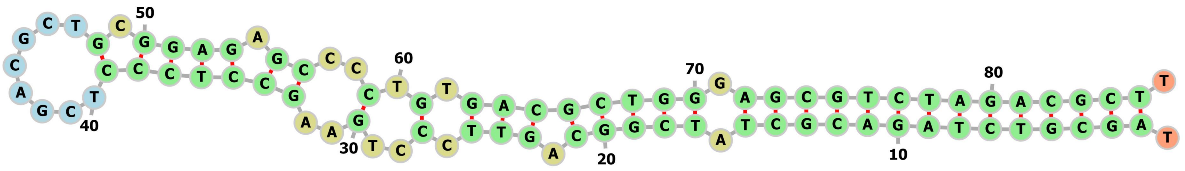

tknovel_miR58 MFE= -21.20 kcal/mol

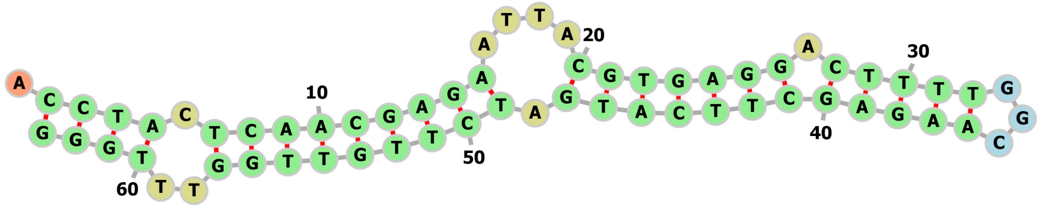

tknovel_miR59 MFE= -36.20 kcal/mol

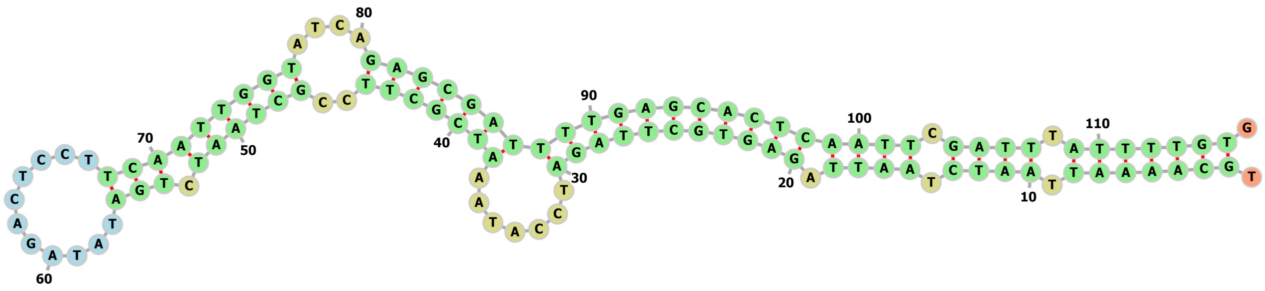

tknovel_miR60 MFE= -83.20 kcal/mol

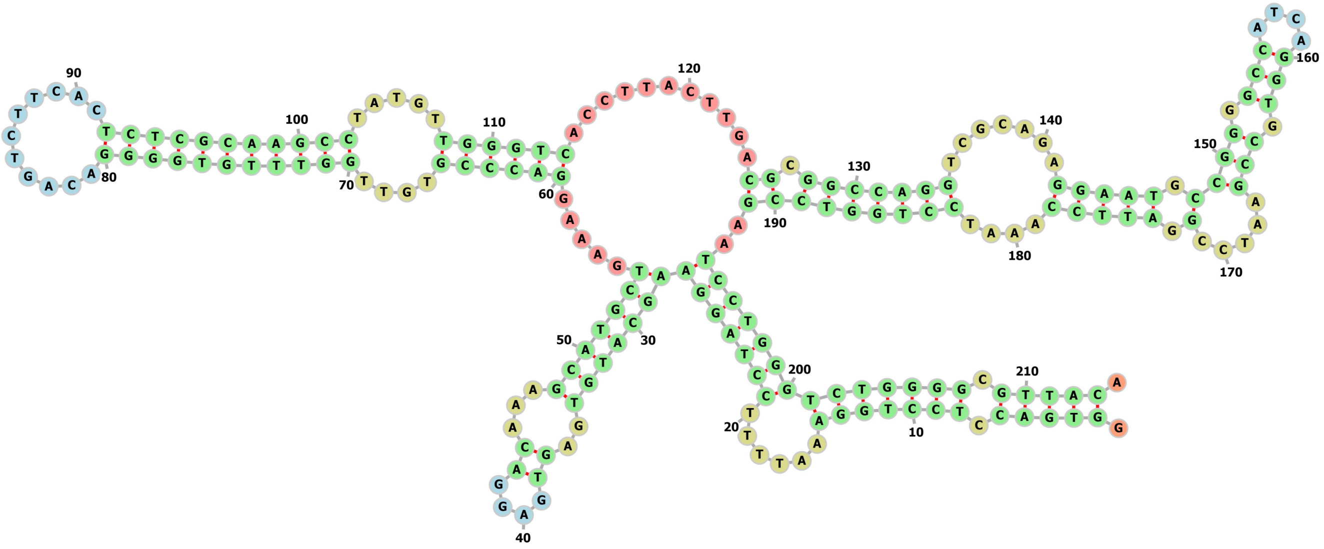

tknovel_miR61 MFE= -50.70 kcal/mol

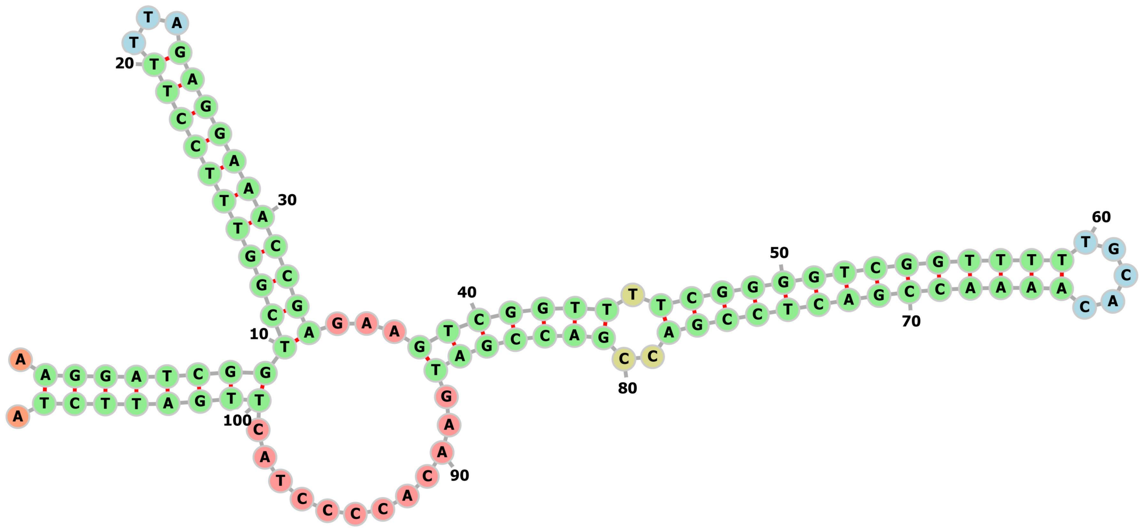

tknovel_miR62 MFE= -66.60 kcal/mol

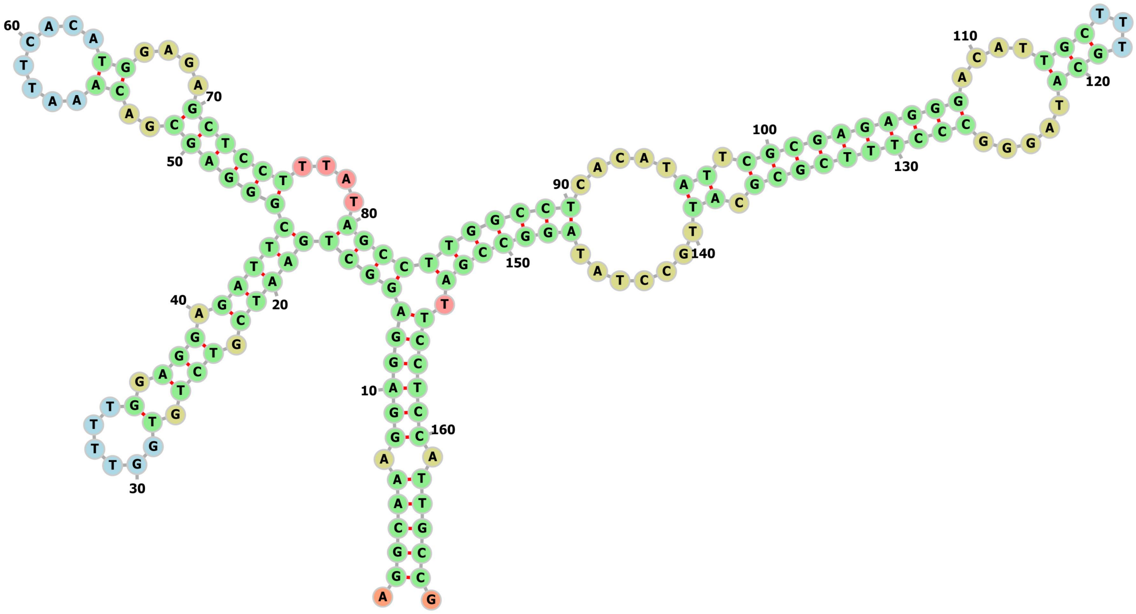

tknovel_miR63 MFE= -56.10 kcal/mol

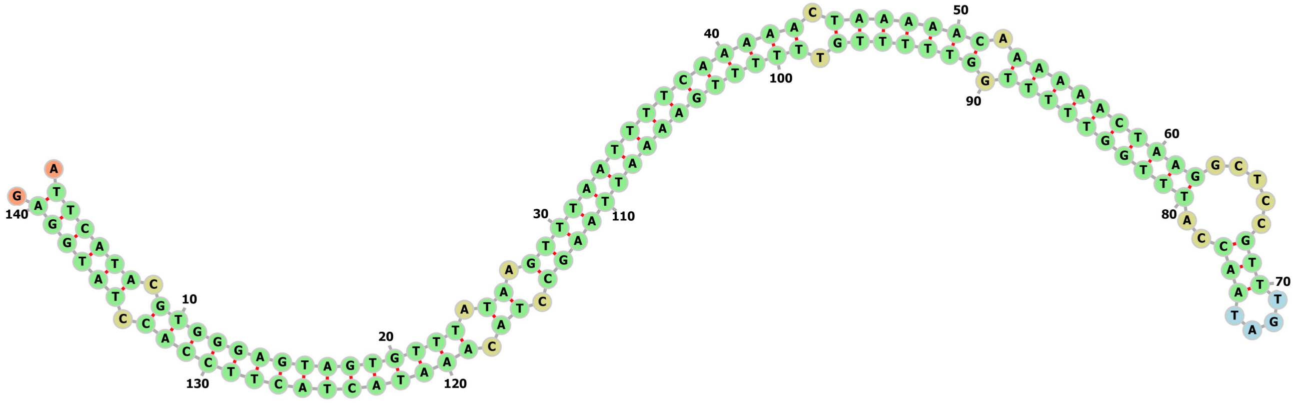

tknovel_miR65 MFE= -50.30 kcal/mol

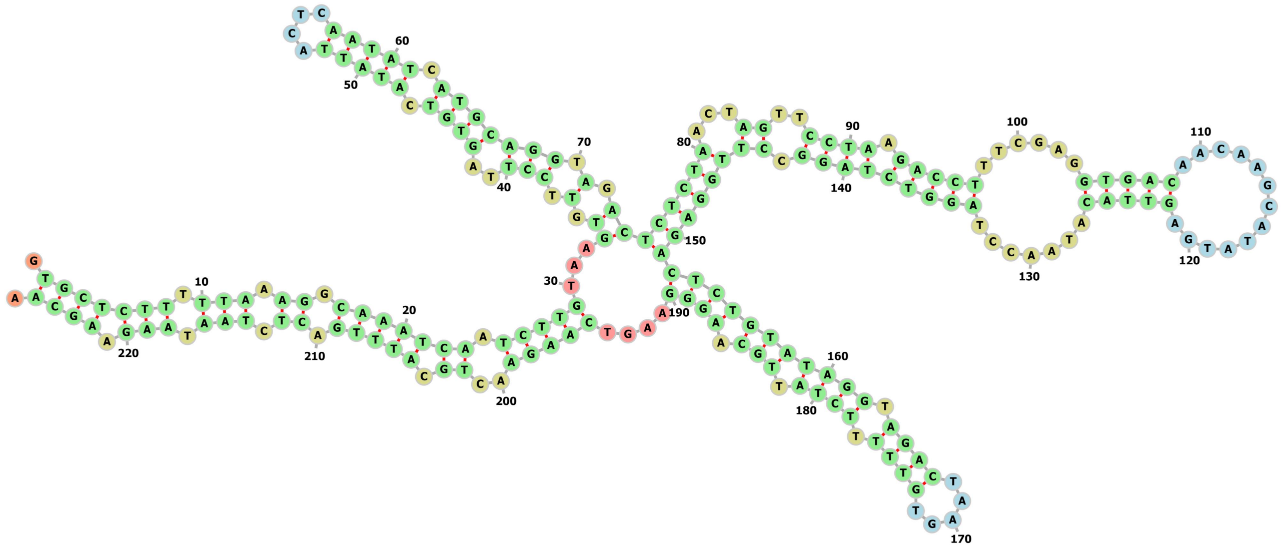

tknovel_miR66 MFE= -47.70 kcal/mol

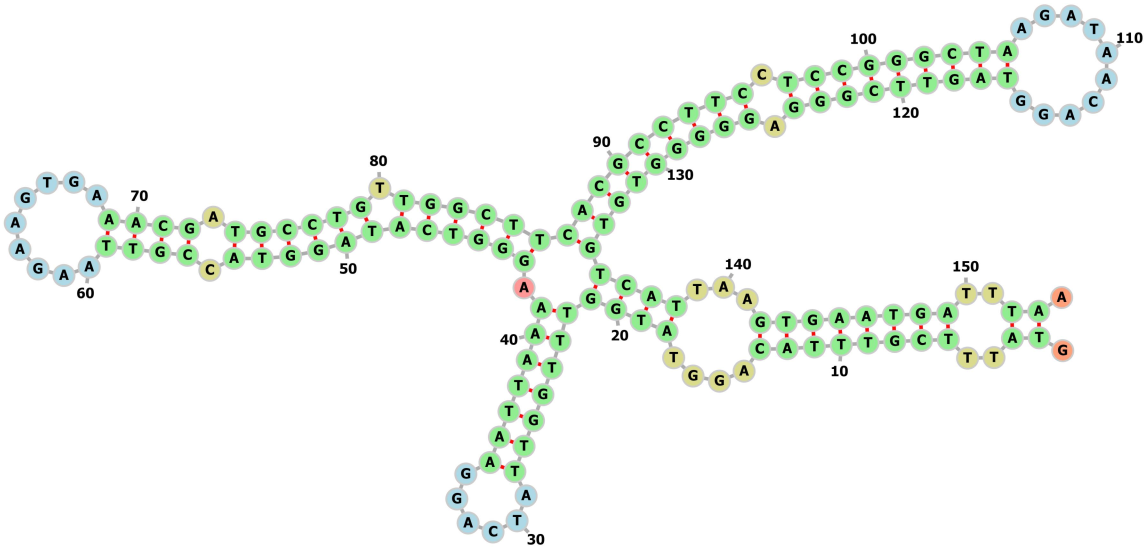

tknovel_miR67 MFE= -29.40 kcal/mol

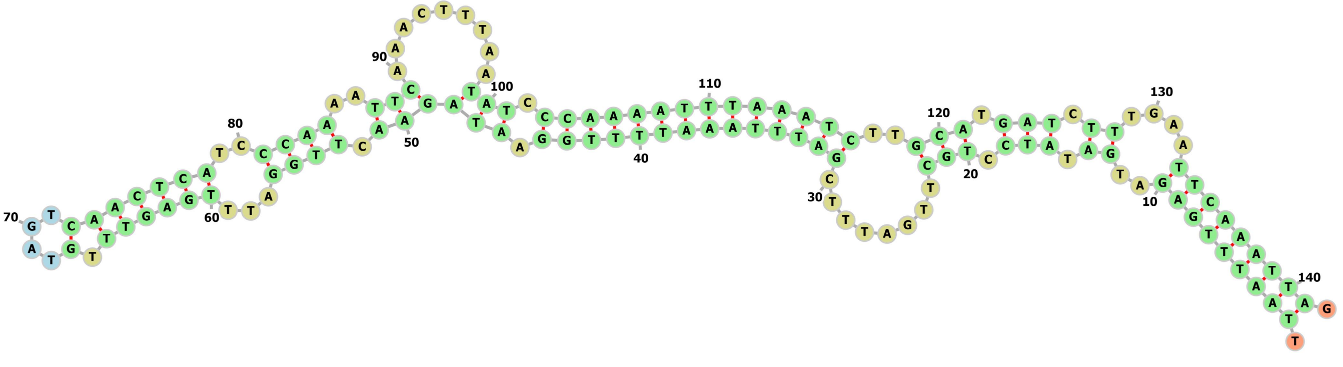

tknovel_miR69 MFE= -20.50 kcal/mol

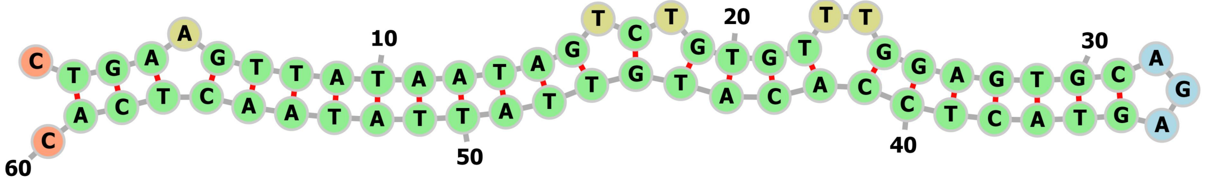

tknovel_miR71 MFE= -47.10 kcal/mol

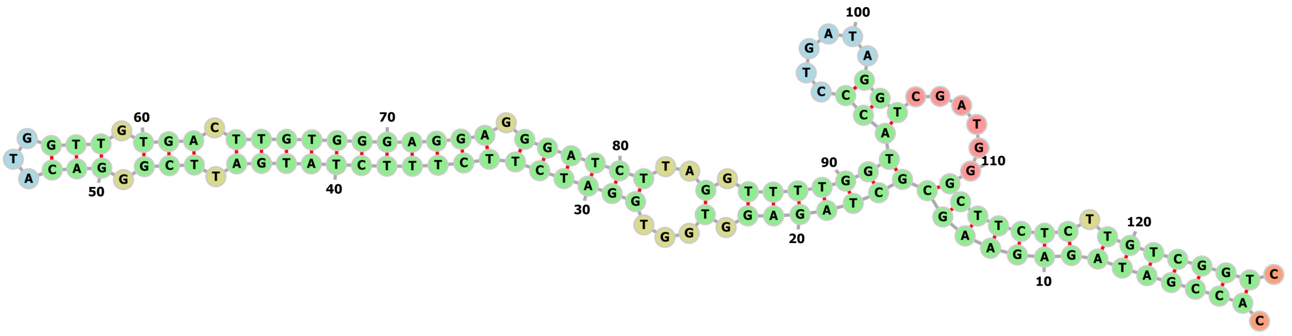

tknovel_miR72 MFE= -66.60 kcal/mol

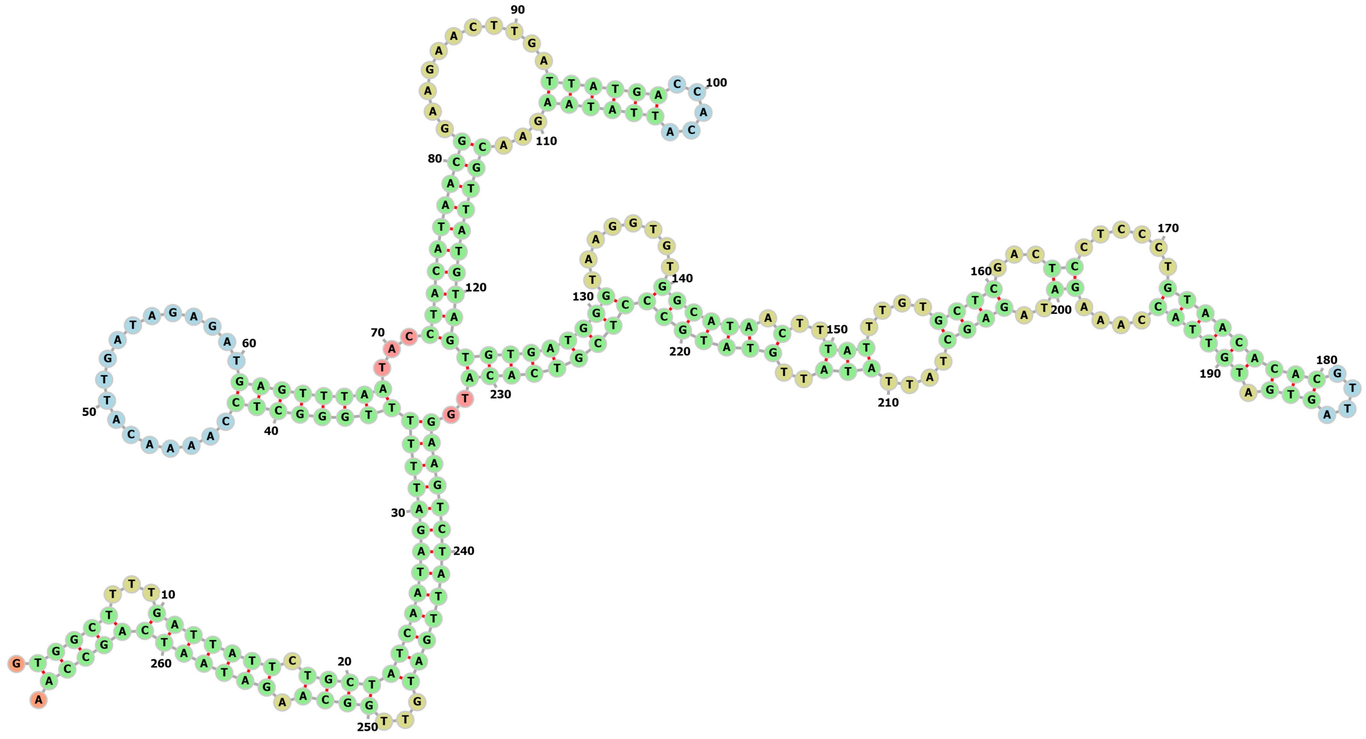

tknovel_miR73 MFE= -51.60 kcal/mol

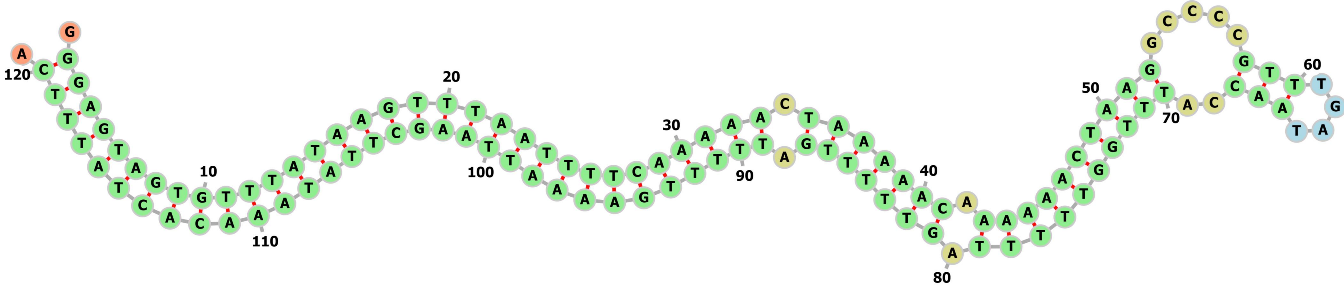

tknovel_miR75 MFE= -92.00 kcal/mol

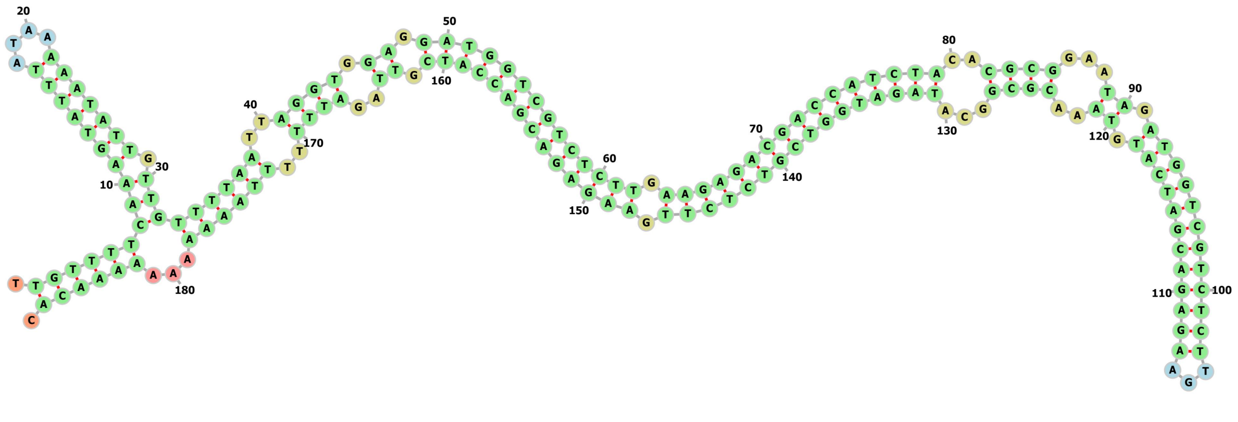

tknovel_miR80 MFE= -24.10 kcal/mol

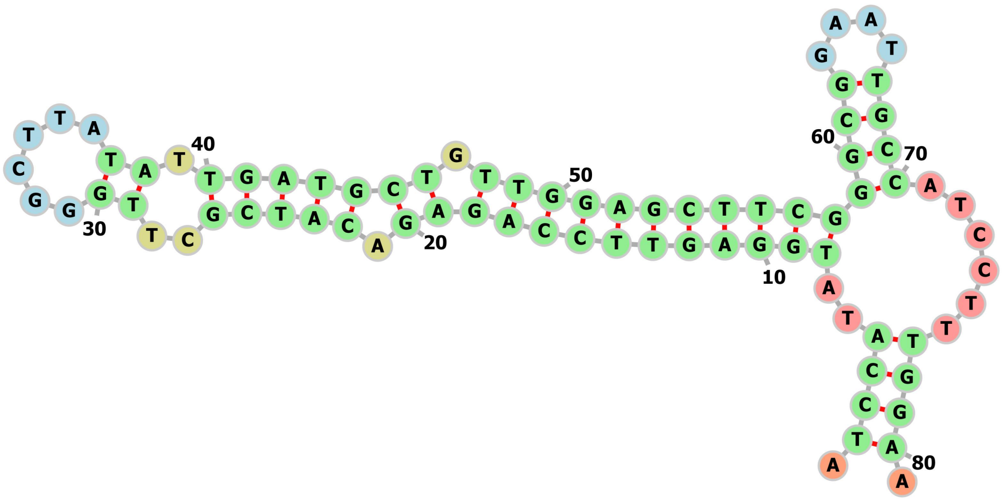

tknovel_miR81 MFE= -44.60 kcal/mol

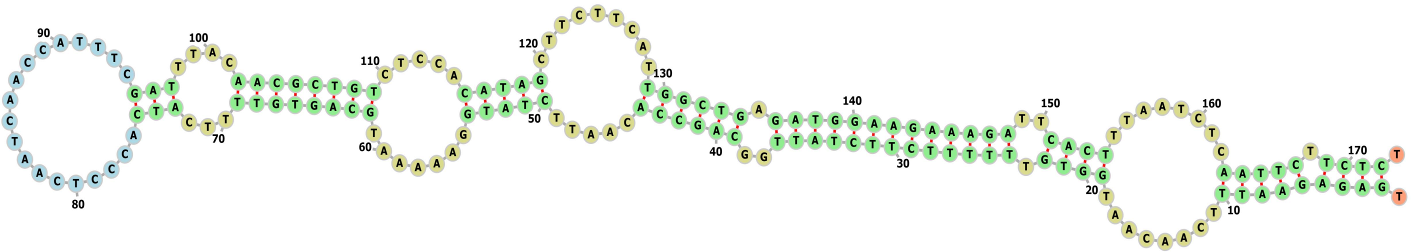

tknovel_miR82 MFE= -59.80 kcal/mol

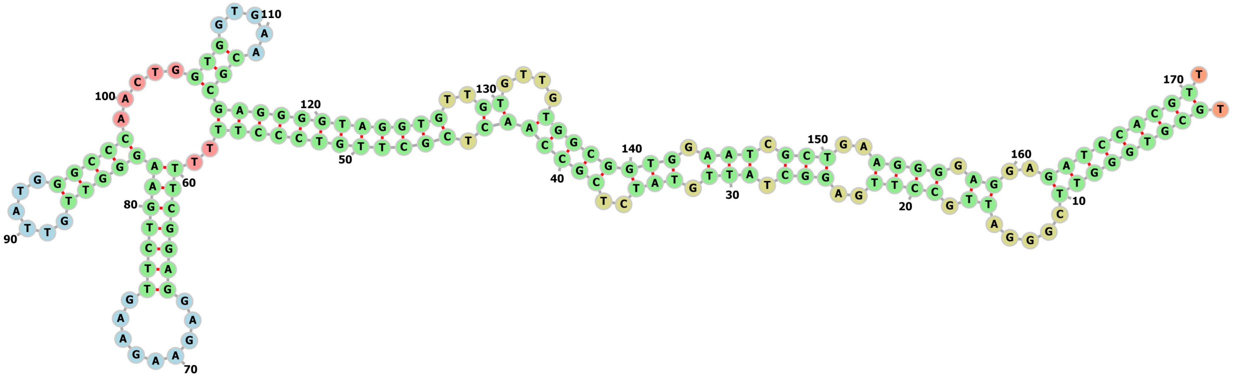

tknovel_miR83 MFE= -64.20 kcal/mol

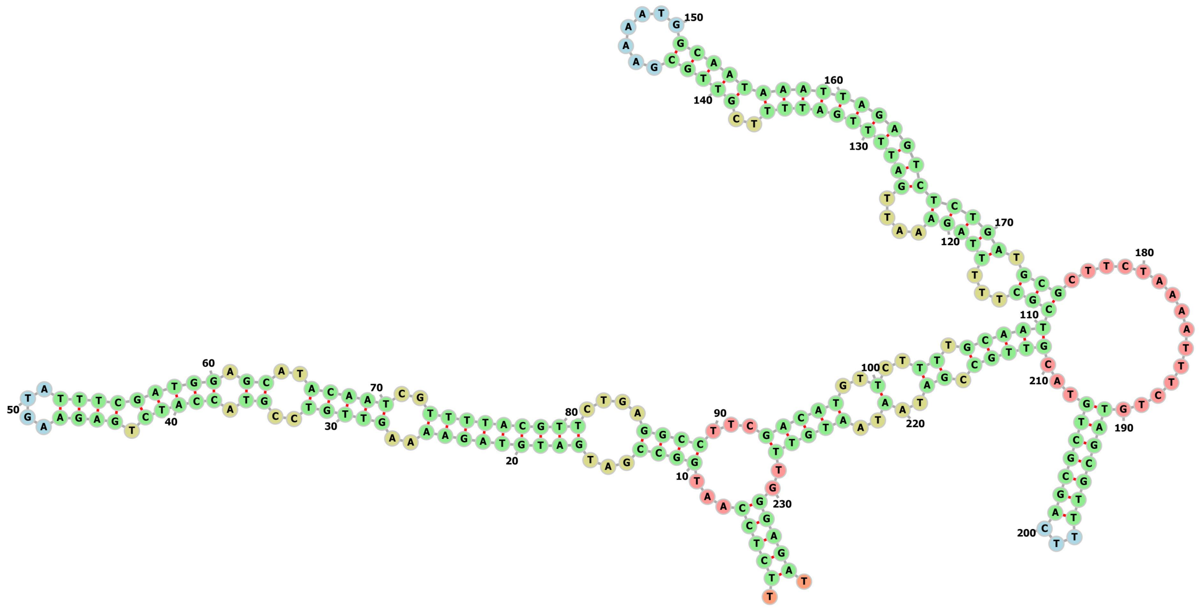

tknovel_miR85 MFE= -33.50 kcal/mol

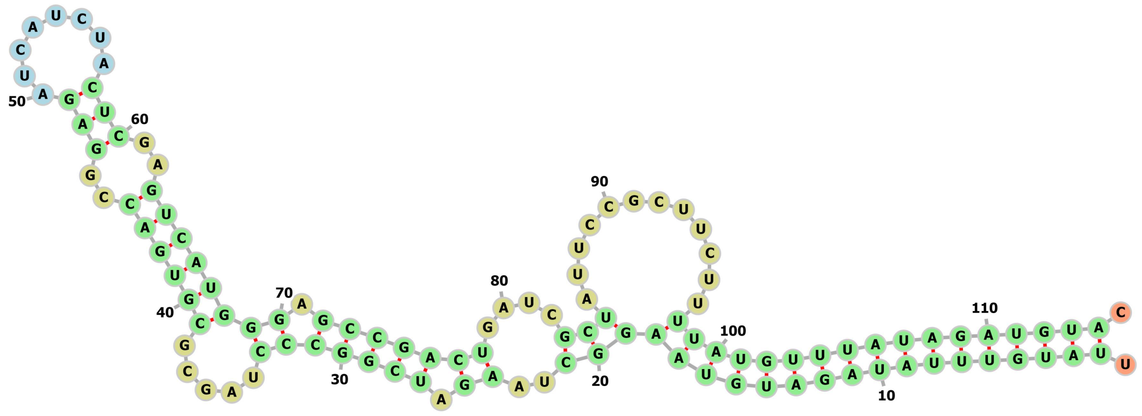

tknovel_miR90 MFE= -57.80 kcal/mol

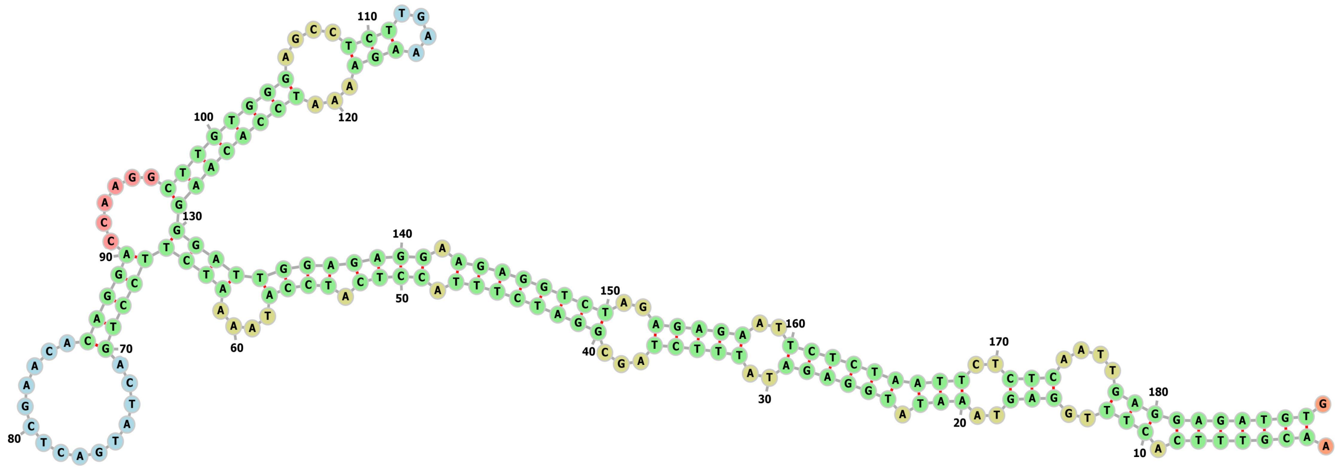

tknovel_miR92 MFE= -35.60 kcal/mol

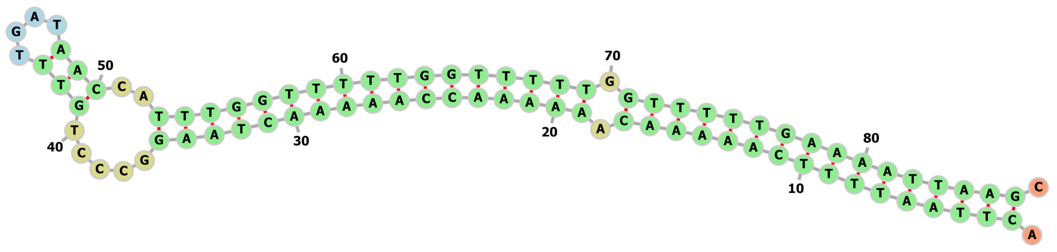

tknovel_miR93 MFE= -37.80 kcal/mol

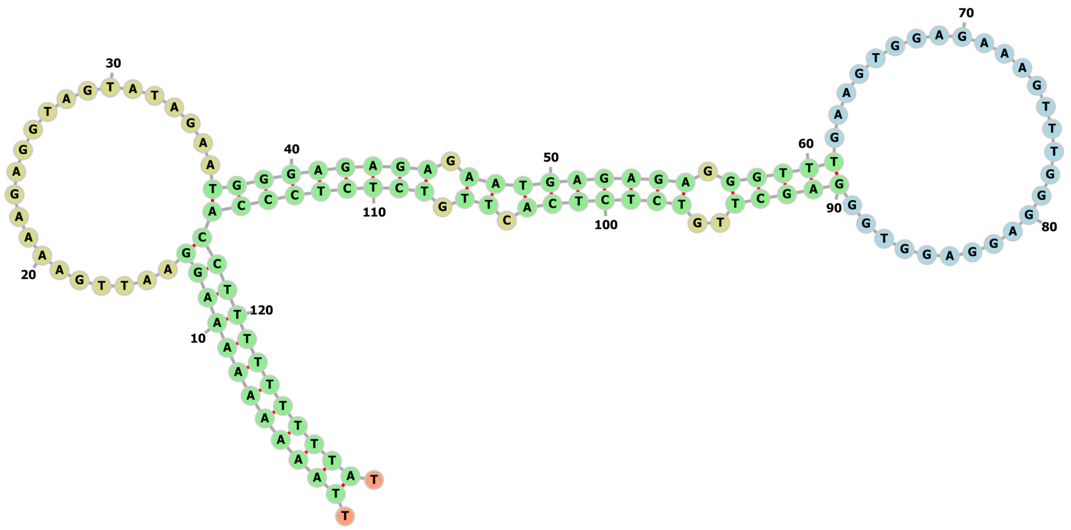

tknovel_miR96 MFE= -51.70 kcal/mol

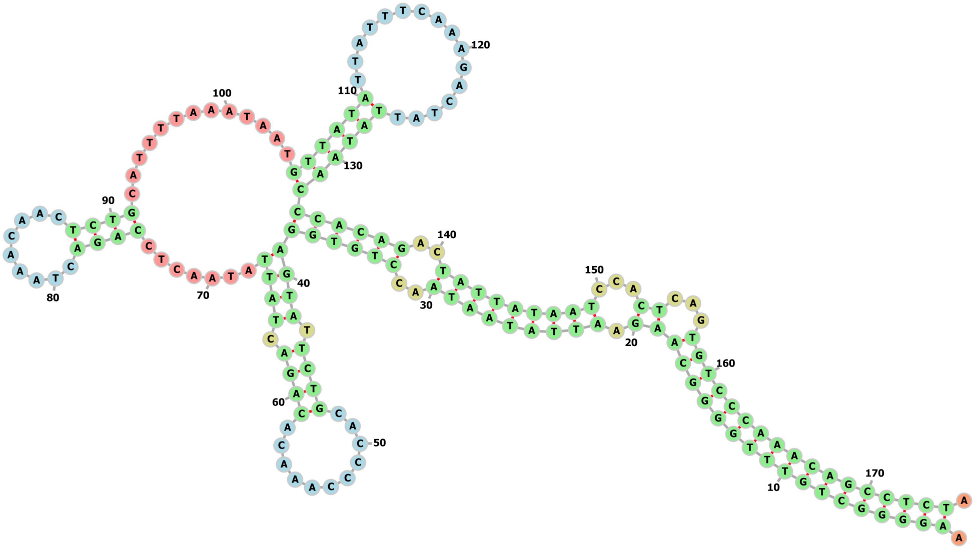

tknovel_miR97 MFE= -62.20 kcal/mol

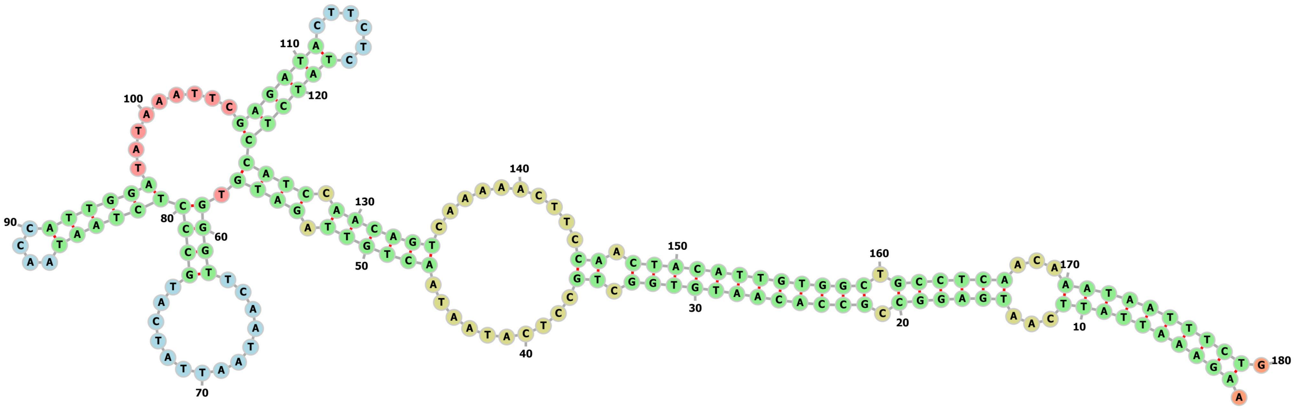

tknovel_miR98 MFE= -49.70 kcal/mol

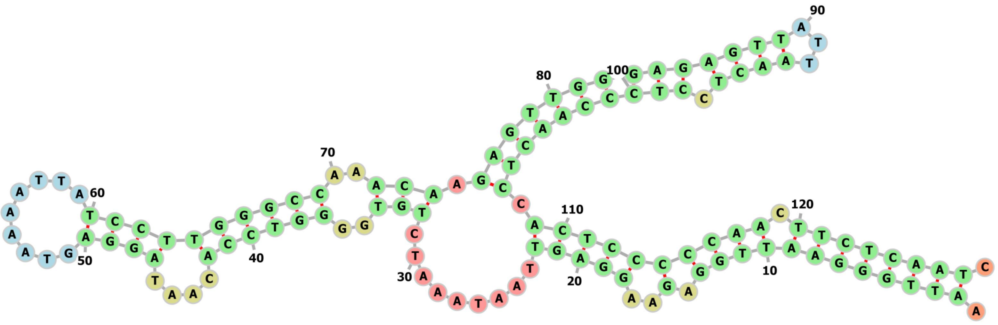

tknovel_miR99 MFE= -27.40 kcal/mol

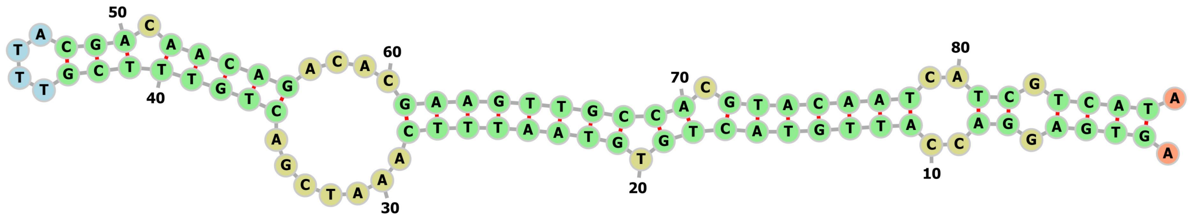

tknovel_miR102 MFE= -61.60 kcal/mol

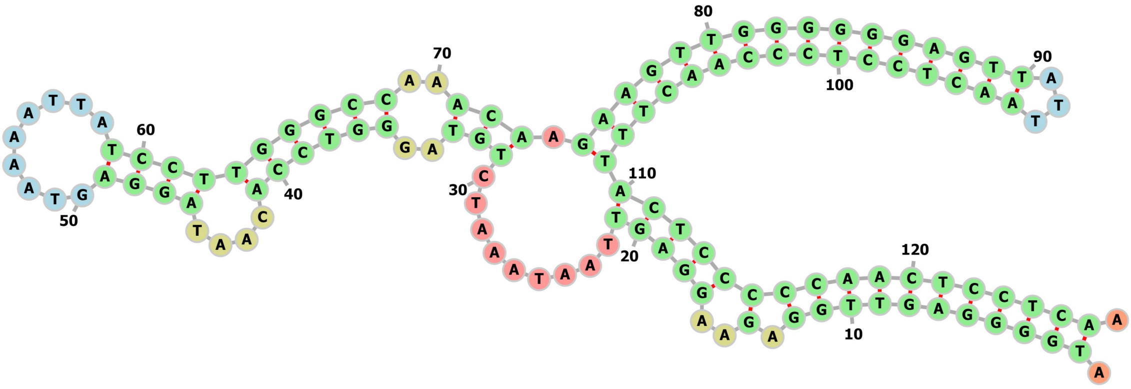

tknovel_miR103 MFE= -56.20 kcal/mol

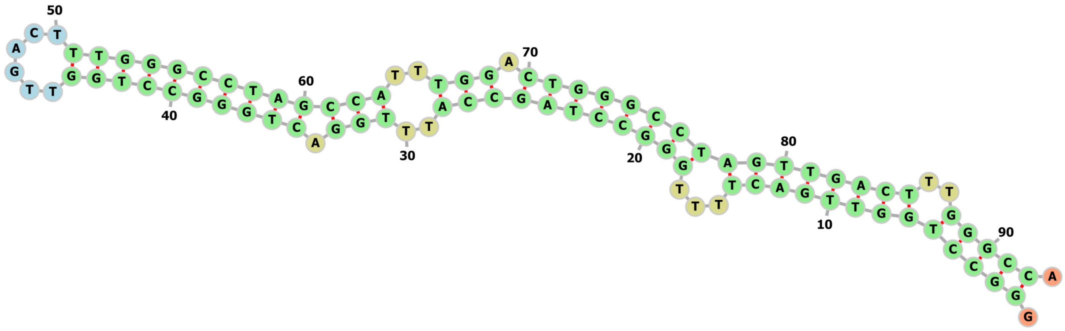

tknovel_miR107 MFE= -39.00 kcal/mol

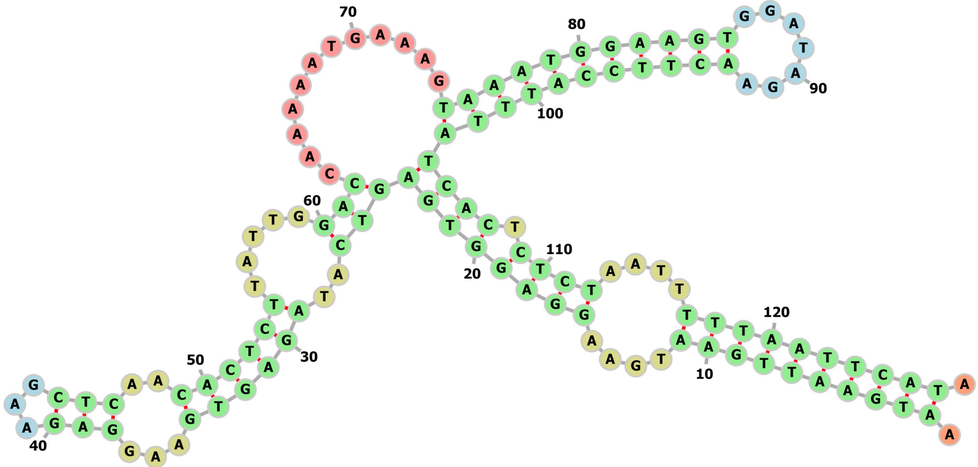

tkmiR156k_2 MFE= -90.30 kcal/mol

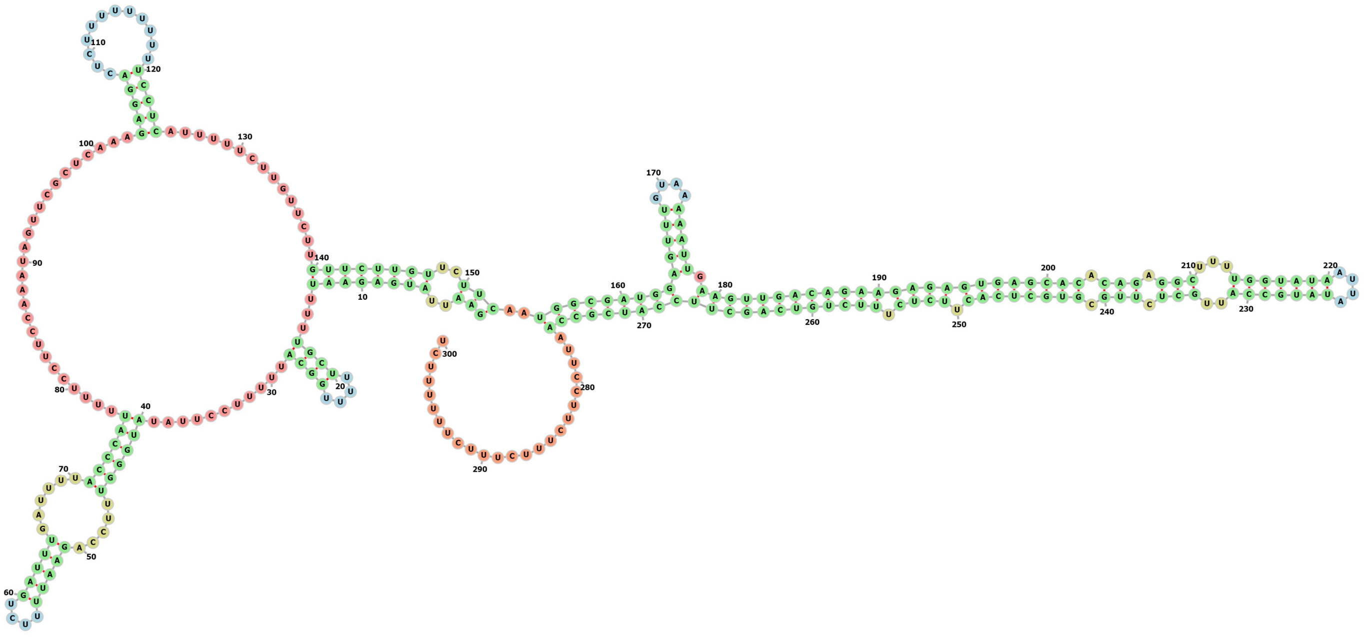

tkmiR156c MFE= -48.60 kcal/mol

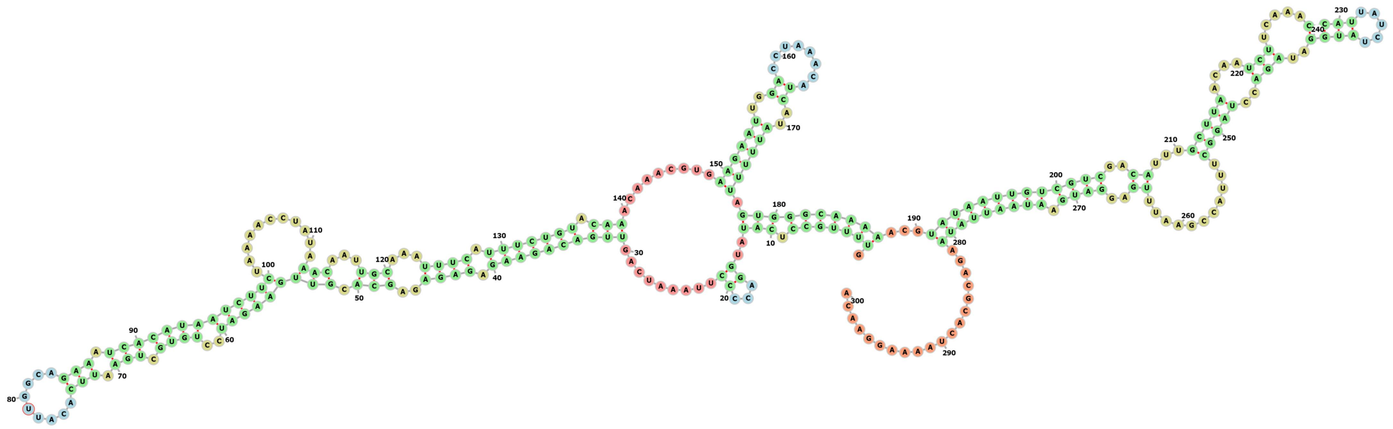

tkmiR156_2 MFE= -76.30 kcal/mol

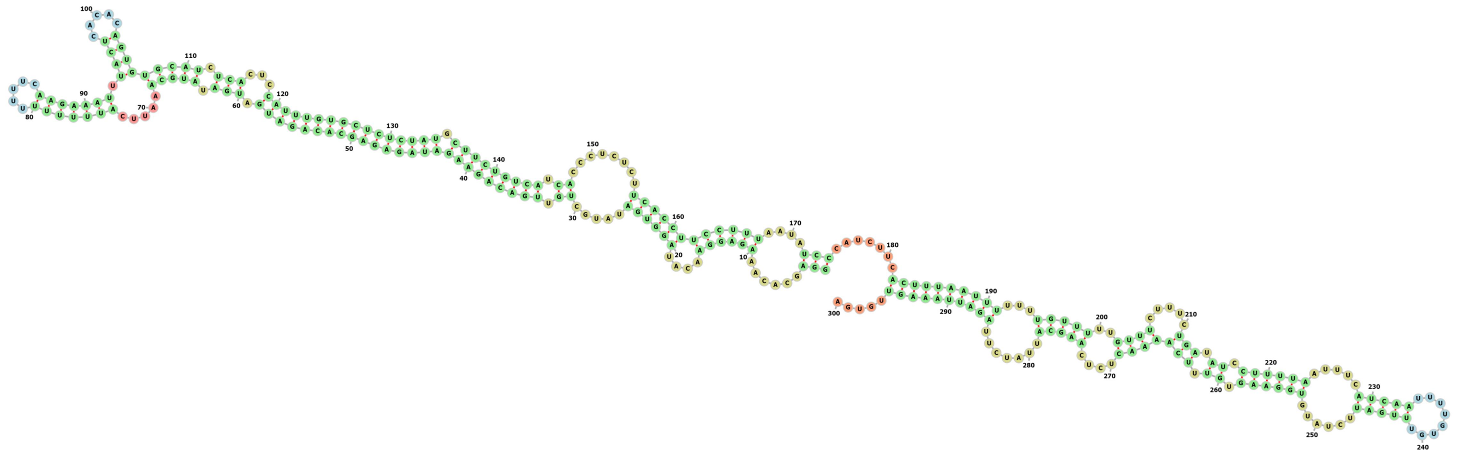

tkmiR396b-5p MFE= -67.30 kcal/mol

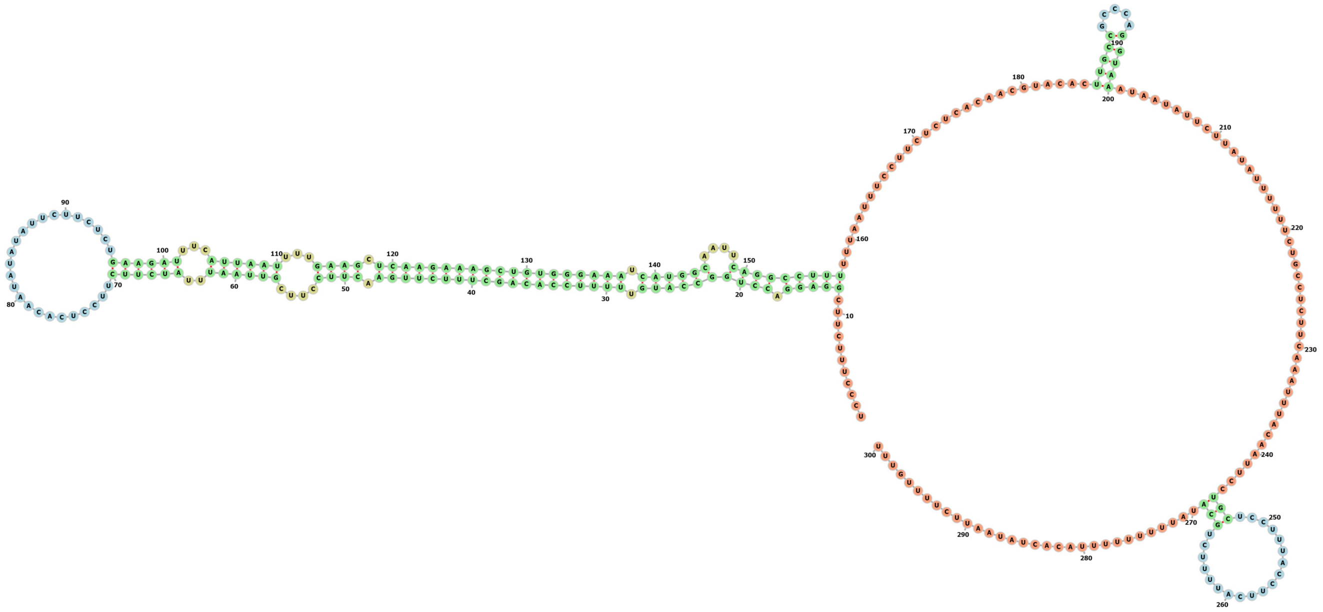

tkmiR396a-3p_5 MFE= -31.40 kcal/mol

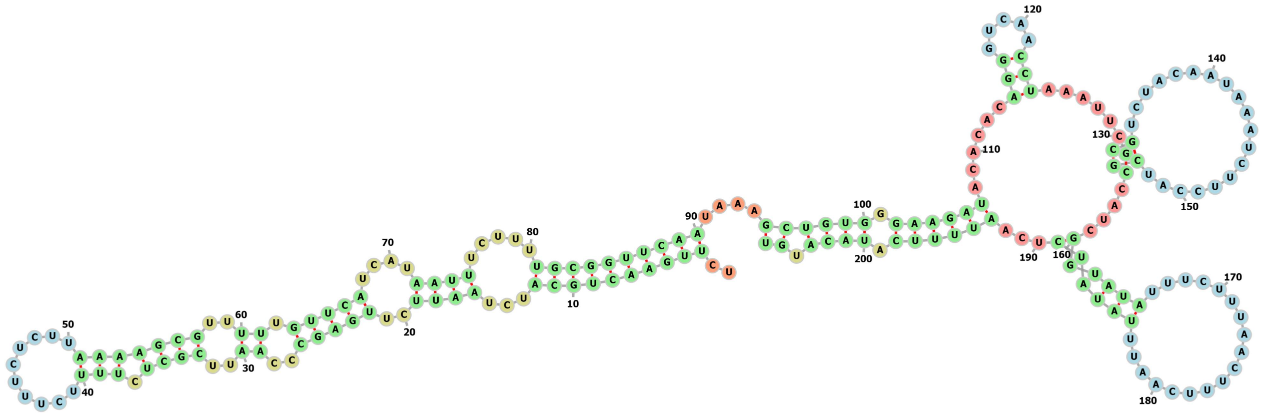

tkmiR396f_1 MFE= -70.10 kcal/mol

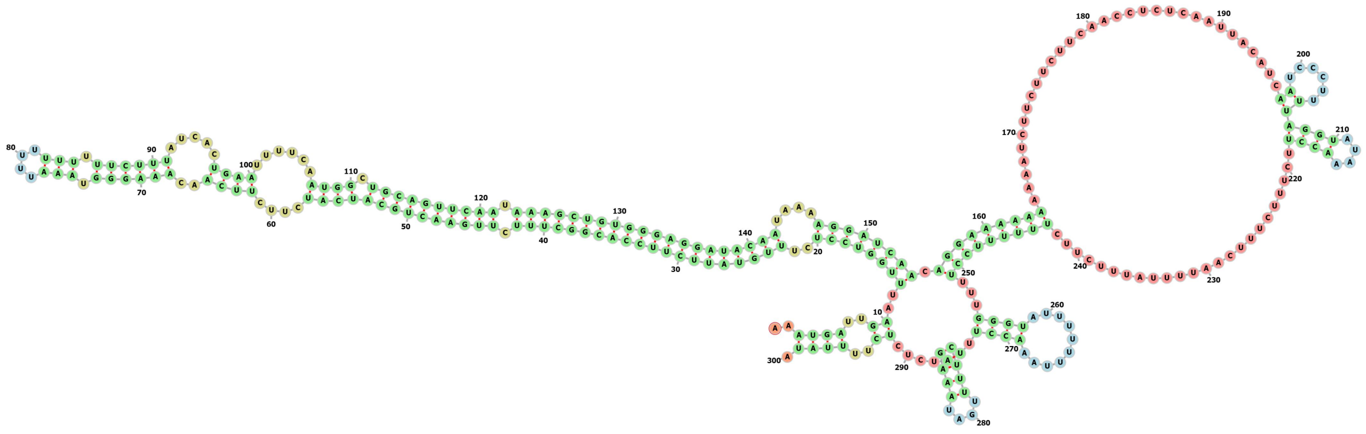

Supplement: Supplementary file 2 — Additional file 2. The stem-loop structures for miRNA. [file 12864_2023_9178_MOESM2_ESM.pdf]
